# Synthesis of point-modified mRNA

**DOI:** 10.1093/nar/gkac719

**Published:** 2022-09-05

**Authors:** Jasmin Hertler, Kaouthar Slama, Benedikt Schober, Zeynep Özrendeci, Virginie Marchand, Yuri Motorin, Mark Helm

**Affiliations:** Institute of Pharmaceutical and Biomedical Sciences, Johannes Gutenberg-Universität, Staudinger Weg 5, D-55128 Mainz, Germany; Institute of Pharmaceutical and Biomedical Sciences, Johannes Gutenberg-Universität, Staudinger Weg 5, D-55128 Mainz, Germany; Institute of Pharmaceutical and Biomedical Sciences, Johannes Gutenberg-Universität, Staudinger Weg 5, D-55128 Mainz, Germany; Institute of Pharmaceutical and Biomedical Sciences, Johannes Gutenberg-Universität, Staudinger Weg 5, D-55128 Mainz, Germany; IMoPA UMR7365 CNRS-UL, BioPole Université de Lorraine, Vandœuvre-lès-Nancy, France; IMoPA UMR7365 CNRS-UL, BioPole Université de Lorraine, Vandœuvre-lès-Nancy, France; Epitranscriptomics and RNA Sequencing (EpiRNA-Seq) Core Facility, UMS2008 IBSLor (CNRS-UL)/US40 (INSERM), Université de Lorraine, Vandœuvre-lès-Nancy, France; Institute of Pharmaceutical and Biomedical Sciences, Johannes Gutenberg-Universität, Staudinger Weg 5, D-55128 Mainz, Germany

## Abstract

Synthetic mRNA has recently moved into the focus of therapeutic and vaccination efforts. Incorporation of modified nucleotides during *in vitro* transcription can improve translation and attenuate immunogenicity, but is limited to triphosphate nucleotides which are accepted by RNA polymerases, and their incorporation is either random or complete. In contrast, site-specific modification, herein termed ‘point modification’ in analogy to point mutations, holds significant technical challenge. We developed fundamental techniques for isolation of long, translatable and internally point-modified mRNAs. Enabling concepts include three-way-one-pot splint ligations, and isolation of mRNA by real-time elution from agarose gels. The use of blue light permitted visualization of mRNA in pre-stained gels without the photochemical damage associated with the use of hard UV-radiation. This allowed visualization of the mRNA through its migration in the agarose gel, which in turn, was a prerequisite for its recovery by electroelution into precast troughs. Co-eluting agarose particles were quantified and found to not be detrimental to mRNA translation *in vitro*. Translation of EGFP-coding mRNA into functional protein was quantified by incorporation of ^35^S-labelled methionine and by in-gel EGFP fluorescence. This enabled the functional analysis of point modifications, specifically of ribose methylations in the middle of a 1371 nt long mRNA.

## INTRODUCTION

Synthetic mRNA has recently moved from side-line applications in fundamental research into the focus of vaccination and therapeutic applications (1,2). With the central dogma of molecular biology in mind, any benefit resulting from a protein, may in principle also be supplied as nucleic-acid-encoded information, to be translated into protein in the body. After an initial boost in gene therapy, DNA as a carrier of that information suffered serious drawbacks (3). Indeed, mRNA can overcome a major concern for conventional DNA-based therapy, namely genomic integration. For fundamental research as well as for therapy, the transient nature of mRNA expression is both a drawback and a boon, and therefore the object of intense research. Given the penchant of long RNAs (4) for degradation, the delivery was and is a major hurdle, typically being approached by packaging RNA into various types of liposomes and similar nanoparticle formulations (5,6). A most timely application is the use of mRNA as a vaccine, where the uptake of antigen-encoding mRNA into monocytes is a central step (7). Once the mRNAs are delivered into monocytes, in particular into dendritic cells, they are subject to molecular inspection for structural characteristics of microbial/viral infection by various endosomal and cytoplasmic PRRs (pattern recognition receptors) (8). Upon positive recognition, they can trigger cascading pro-inflammatory cytokine emission and additional downstream antiviral responses. Of note, an inspection of HIV genomic RNA identified a ribose methylation which, along these lines, was determined to impede cellular immune response (9). In contrast, in certain therapeutic intentions, PRR signalling may be desired, e.g. for certain vaccination schemes or better be circumvented (10). Several groups found *in vitro* transcripts to be recognized by PRRs (11–13). At the onset of a new field of research, the seminal studies by Karikó and Weissmann reported in 2005 that incorporation of modified nucleosides into mRNAs affected immune response as well as translation efficacy. Upon incorporation into mRNA *via* their triphosphates by T7 RNA polymerase, 2-thiouridine (s^2^U), 5-methyluridine (m^5^U), 5-methylcytidine (m^5^C), N6-methyladenosine (m^6^A) and pseudouridine (Ψ) led to decreased immunogenicity by reducing signalling of TLRs (14), while m^5^C and Ψ-containing RNA simultaneously augmented protein expression (10). Among the most recently developments are the use of N1-methyl pseudouridine (m^1^Ψ), which reportedly outperformed pseudouridines in cell-based and mice experiments (15–17), contributing to the Karikó ‘paradigm’ in this growing field of research.

The current widespread use of mRNA vaccines builds on proof-of-concept studies in passive as well as in active immunization. Pardi *et al.* described passive immunization *via* the administration of m^1^Ψ-containing mRNAs encoding the light and heavy chains of anti-HIV-1 antibody VRC01, was able to protect humanized mice against intravenous HIV challenge (18–20). An active immunization approach was studied in rabbits and rhesus macaques (21). Another example of the use of modified mRNA is the generation of immunity against Zika virus in mice and rhesus macaques (22,23). Very recently, facing the outbreaking pandemic SARS-CoV-2 that appeared in late 2019, several mRNA-based vaccination programs were launched of which two active vaccines emerged that now constitute major weapons in the fight against the pandemic.

In all the above, an important facet of RNA modification research is missing. Recent results from fundamental research clearly point out strong impact of naturally occurring RNA modifications on essentially all aspects of translation (24,25) as well as on immunogenicity (26,27). This applies most prominently to m^1^Ψ, a uridine surrogate used in the mRNA vaccines by BioNTech/Pfizer and Moderna. Another potentially very important type of natural RNA modifications reported to be occurring in mRNAs are ribose methylations (2′-O-Me, N_m_) (28–30). Certain sequences including a G_m_ residue are known to have an antagonistic effect on the activation of Toll like receptor 7 (31–34) and therefore are important suppressors of RNA-induced PRRs activation (8). Making use of these effects by the hitherto discussed approaches is rendered difficult to impossible for two reasons. Firstly, not all relevant RNA modifications, such as ribose methylations are amenable to T7-catalyzed incorporation into RNA *via* their triphosphates. Secondly, naturally occurring modifications in coding RNA are typically site-specific, i.e. their impact cannot be controlled if they occur as pervasive, complete modification, or in the random distribution patterns that are created by T7 RNA polymerase.

### Site specific modification in synthetic mRNA

Options are quite limited to test the impact of single site modifications in full-length mRNA on e.g. translation. Some studies have been conducted with workaround solutions, often including mRNA or minimal surrogates thereof, which lack features relevant for expression in living cells (35). Meyer *et al.* explored the effect of a single m^6^A on mRNA translation initiation promotion under stress conditions. This was achieved using N6-methyladenosine 5′-monophosphate (m^6^AMP) and a promoter that starts with an A at the transcription initiation site (36). The Puglisi group used a 30 nts long mRNA surrogate in biophysical studies revealing variegated impact of ribose methylations at different positions of a given codon (37). This observation was also made by the Erlacher group, using short mRNAs coding for a 19-amino-acid peptide in both prokaryotic *in vitro* translation systems (35,38). The hitherto most advanced studies from the Erlacher group used splint ligation to join two RNA fragments into mRNAs that give rise to 15 kDa long proteins (39,40) upon transfection into HEK cells.

Splint ligation typically includes a combination of RNA *in vitro* transcription and a chemically synthesized short RNA containing the desired modification. The correct orientation of the fragments is ensured by a splint DNA, which hybridizes the ends of the RNA fragments, directing a DNA or RNA ligase (e.g. RNA ligase II) activity in joining 5′ and 3′ ends (41).

However, hampered by the characteristics of RNA synthesis by phosphoramidite chemistry, the splint ligation approach of joining two RNA fragments necessarily limits the site of modification to the first ∼30–40 nucleotides from either extremity (39,40). Conceivably, one might consider synthesizing an mRNA by two successive ligations, with the chemically modified fragment ending up in the middle, and thus providing access to point-modifications at any chosen site in long RNAs. However, procuring the necessary amounts of starting material for such an endeavour has so far been prohibitive because purification of full-length mRNA incurs heavy losses, especially during elution from gel after electrophoresis. This turns out to be an important yet underestimated step in the manufacturing process of synthetic mRNA, necessary to remove starting material, side products, and residual contaminants. Remarkably, aside from one much-observed work by Karikó and Weissmann (42), little literature is available on the impact of purification, (e.g. by HPLC) on mRNA performance. The recovery of mRNA becomes more challenging when synthesized by ligation, as reliable size separation becomes more important for isolation of ligated product close in size to initial substrate fragment. So far, gel electrophoresis is commonly used for this purpose (38,43–46) and other techniques like magnetic bead-based poly (A) purification remain scarce (35). However, extraction of RNA from gels of various types and concentrations is a lasting problem in the field, featuring multiple problems especially for upscaling the production of large RNAs.

In this paper, we report a procedure enabling successful three-way-one-pot splint ligation, purification and translation of long (>1300 nts) mRNA carrying a point-modification in its middle, remote from any extremity. As an elemental step, we also present a purification method allowing facile recovery of full-length mRNA from an agarose gel by what we termed ‘real-time’ gel elution. We characterize the ensuing agarose particle content and show by in-gel detection of EGFP protein fluorescence that it is compatible with translation. Comparison of point-modified mRNA carrying internal ribose methylations precisely recapitulated strong intra-codon positional effects on *in vitro* translation of EGFP.

## MATERIALS AND METHODS

### RNA synthesis by *in vitro* transcription

Circular plasmid pUC57 vector (vector map can be found in supplementaries) containing the integrated sequence of the respective RNA fragment (GenScript, USA) under a T7-promotor was either linearized by the restriction enzymes BamHI and SacII for EGFP constructs, MbiI and BcuI for mScarlet-I constructs (Thermo Fisher Scientific, Germany) and by Eco53kI for SARS-CoV-2 Nsp13 constructs respectively (New England Biolabs, Germany). Buffers and enzyme concentrations were used according to the manufacturers protocol and reactions were incubated overnight at 37°C. The reaction mixture was extracted two times with chloroform and phenol (TE-buffered, Carl Roth) at a ratio of 2:1:1, then twice with the same volume chloroform and once with the same volume diethyl ether to remove residual phenol traces. During each step the phases were well homogenized and then separated by centrifugation. To recover the DNA, ammonium acetate/ethanol precipitation was carried out by addition of 1/10 volume 5 M ammonium acetate (Merck-Millipore, Germany), 1 μl glycogen (5 mg/ml, Thermo Fisher Scientific, Germany) and 2 vol. 100 % ethanol (Carl Roth, Germany). The samples were incubated at −80°C for 1 h or at −20°C overnight. The DNA pellet was collected by centrifugation at 12 000 g at −4°C for 45 min, washed with 75% ethanol and again centrifuged at 12 000 g at −4°C for 15 min. The resulting DNA pellet was dissolved in ultrapure water.

The *in vitro* transcription with in-house T7 RNA polymerase was conducted in 40 mM Tris–HCl (pH 8.1), 1 mM Spermidine, 10 mM DTT and 0.01% Triton X-100 as well as final concentration of 30 mM MgCl_2_ and 2.5 μg/ml bovine serum albumin (Thermo Fisher Scientific). The NTP’s (GTP, ATP, CTP, UTP) were added to a final concentration of 5 mM. A typical transcription was performed with 10 μg DNA in a total volume of 200 μl. Alternatively, HiScribe T7 High Yield RNA Synthesis Kit (New England Biolabs, Germany) was used according to the manufacturer's protocol.

For transcripts that required a 5′-phosphate for subsequent ligation, the final concentration of GTP was reduced to 2 mM and GMP was added to 16 mM final concentration (47). The DNA Template was digested by adding 2 U DNase I (Thermo Fisher Scientific, Germany) per 1 μg DNA followed by incubation at 37°C for 30 minutes. Transcription products were purified with MegaClear™ Kit from Thermo Fisher Scientific. Modified and unmodified short RNA fragments (fragment #2) were chemically synthesized by IBA (Germany) or Biomers (Germany). Sequences are given in the supplement.

### RNA ligation

Commercial oligonucleotides were 5′ phosphorylated prior to ligation with T4 polynucleotide kinase (Thermo Fisher Scientific, Germany). 100 pmol of oligonucleotide were incubated for 1 h at 37°C in 1× buffer A as well as 5 mM DTT (Invitrogen, Germany) and 5 mM ATP together with 90 U enzyme in a total volume of 40 μl. After phosphorylation, equimolar amounts of the *in vitro* transcribed fragments were added to this reaction, as well as 1× T4 RNA Ligase 2 buffer (New England Biolabs, Germany). The amount of substance of the respective DNA splint was 2% less than the respective RNA fragments. This mixture was incubated for 4 min at 75°C and allowed to cool down to room temperature over 15 minutes. Then the ligation was carried by the addition of 1 U of T4 RNA Ligase 2 (New England Biolabs, Germany) per 0.8 μg of RNA. For the respective reaction: 57 U T4 RNA ligase 2 in a total volume of 100 μl. The reaction was incubated overnight at 16°C. DNA splint was digested with 2 U of DNase I (Thermo Fisher Scientific, Germany) per 1 μg DNA for 30 min at 37°C.

### Analysis (concentration and purity determination)

The respective DNA or RNA samples were mixed with denaturing loading dye and applied to an agarose gel containing 1 % agarose in 1× TBE buffer. The gel was either pre-stained with SYBR^®^Gold (Thermo Fisher Scientific, Germany), diluted 1:10,000 during pouring of the gel, or post-stained with 1× GelRed (from 3× Concentrate; BioTrend, Germany) for 20 min. The running of the gel was stopped when bromphenol blue migrated almost to the end of the gel. The results were visualized on a Typhoon 9600 trio+ (GE Healthcare, United Kingdom). The settings were as indicated in Table 1.

**Table 1.** Typhoon settings

Dye	Laser excitation (nm)	Emission filter (nm)
GelRed	532	610 BP 30
SYBR^®^Gold	488/532	520 BP
CY5	633	670 BP 30

The full-length ligation sample was also analysed by 4200 TapeStation from Agilent according to the manufacturer protocol.

The concentration of the samples was determined spectrophotometrically with a NanoDrop 2000 spectrophotometer (PeqLab, Germany).

### Real-time gel elution

For real-time gel elution, the concept of E-Gel CloneWell (Thermo Fisher Scientific) (48) was adapted using Dark Reader Blue Light Transilluminator (Clare Chemical Research, USA) and a conventional agarose gel chamber from PeqLab (Germany). 12-well combs were used where 10 wells were combined with Sellotape. A second comb, prepared in the same way, was placed in the second half of the gel chamber. 1× SYBR^®^Gold (Thermo Fisher Scientific, Germany) was added during the pouring of 1% agarose gel (Biozym, Germany). The ligation reaction (mixed 2:1 with denaturing loading dye containing 90% formamide (Roth, Germany) and 10% 10× TBE were applied into the upper well. Running of the gel was performed at 80 V for 30 min and afterwards at 150 V. All fractions of interest were collected using a 1000 μl pipet. The collection chamber was rinsed between the fractions with 1× TBE buffer. To recover the RNA from eluted fractions, ammonium acetate/ethanol precipitation was carried out by addition of 1/10 volume 5 M ammonium acetate (Merck-Millipore, Germany), 1 μl glycogen (5 mg/ml, Thermo Fisher Scientific, Germany) and 2 vol. 100% ethanol (Carl Roth, Germany). The samples were incubated at −80°C for 1 h or at −20°C overnight. The RNA pellet was collected by centrifugation at 12 000 g at −4°C for 45 min, washed with 75 % ethanol and again centrifuged at 12 000 g at −4°C for 15 min. The resulting RNA pellet was dissolved in ultrapure water. To reduce as much as possible the amount of agarose particles in the recovered RNA preparations, all samples were filtered through 0.2 μm solid phase filters (Nanosep centrifugal device, Pall, USA).

### Low melting agarose

One percent agarose gel was cast using low melting/gelling temperature agarose (Sigma-Aldrich, Germany) and samples were added to the wells after mixing 1:1 with denaturing loading dye. RNA was visualized either by pre-staining the gel with 1× SYBR^®^Gold (Thermo Fisher Scientific) or by post-staining with 1× GelRed (from 3× Concentrate; BioTrend, Germany) for 20 min. Scanning was performed on a Typhoon 9600 trio+ (GE Healthcare, United Kingdom). The desired bands were excised and the extraction of ligation products was performed according to the literature with slight modifications (49). Ultrapure water was used for extraction. The combined aqueous phases were extracted once with saturated phenol and then once with chloroform.

### Glass wool filtration

The glass wool filters were adapted according to previous publications for DNA extraction from agarose gels (50,51). The glass wool (Sigma-Aldrich, Germany) was packed into a 500 μl reaction tube with a hole in the bottom (serving as ‘filter’) and this tube was placed into a 1.5 ml reaction tube, serving as ‘collection tube’.

### Agarose particle tracking

The total volume of the respective purified ligation samples was adjusted to 1 ml, and samples were injected into the sample chamber from a Malvern NanoSight LM10 (Malvern Panalytical, Germany). Scattering of a 532 nm laser allowed detection of the particles in suspension. This was visualized by a microscope with 20x magnification on which an sCMOS camera was mounted. Because of the uneven distribution of size and shape, the number of agarose particles was evaluated by visual inspection rather than by the automated Nanoparticle tracking analysis (NTA) software. Therefore, a predefined number of positions of the camera were chosen at random, and the sizes detected particles were measured using the software GIMP (Freeware, The GIMP-Team). Particles were binned into one of three categories, namely big, medium or small. As limits to separate the categories, the areas of particle profiles were used, such that particles with a 2D profile between 1 μm^2^ and about 17 μm^2^ in the above measurements were classified medium.

To test the degree of agarose particles in relation to RNA sample size, a dilution series with samples containing 16, 4.5 and 1.3 μg RNA respectively was submitted agarose electrophoresis followed by real-time elution with typical recovery rates between 25 and 20%. The 1.3 μg was run 5 times to obtain enough material to pool 1 μg of recovered RNA. The agarose particle content corresponding of 1 μg was then compared among the samples.

### Absolute quantification via LC–MS

For absolute LC–MS/MS analysis in dMRM mode 200 ng μg of the respective RNA sample was digested to nucleoside level by overnight incubation in 25 mM ammonium acetate (pH 7.5, Sigma-Aldrich, Germany) at 37°C with 0.6 U nuclease P1 from *P. citrinum* (Sigma-Aldrich, Germany), 0.2 U snake venom phosphodiesterase from *C. adamanteus* (Worthington, USA), 2 U FastAP (Thermo Fisher Scientific, Germany), 200 ng Pentostatin (Sigma-Aldrich, Germany) and 500 ng Tetrahydrouridine (Merck-Millipore, Germany) were added. For quantification 50 ng of the digested sample were analysed together with 50 ng internal standard (^13^C stable isotope-labelled nucleosides from *S. cerevisiae*, SIL-IS) *via* Agilent 1260 series coupled to a diode array detector (DAD) and Agilent 6460 Triple Quadrupole mass spectrometer equipped with an electrospray ion source. The solvents consisted of 5 mM ammonium acetate buffer (pH 5.3; solvent A) and LC-MS grade acetonitrile (solvent B; Honeywell). The elution started with 100% solvent A, followed by a linear gradient to 8% solvent B at 10 min and 40% solvent B after 20 min. The flow rate was 0.35 ml/min on a Synergi Fusion column (4 μM particle size, 80 Å pore size, 250 × 2.0 mm from Phenomenex). ESI parameters for quantification were as follows: gas temperature 350°C, gas flow 8 l/min, nebulizer pressure 50 psi, sheath gas temperature 350°C, sheath gas flow 12 l/min, capillary voltage 3000 V. The MS was operated in the positive ion mode using Agilent MassHunter software in the dynamic MRM (multiple reaction monitoring) mode. For quantification, a combination of external and internal calibration was applied as described previously (52).

### UV treatment

7 μg of an *in vitro* transcript of full length mRNA were diluted in 100 μl ultrapure water and were transferred to a cuvette. Afterwards, the samples were radiated for 2 h either by 254 nm, blue light from the DarkReader or were kept in the dark at room temperature as negative control. Prior to digestion for LC–MS analysis the samples were precipitated as described above and digested as follows: 5.6 μg of the respective RNA sample was digested to nucleoside level by overnight incubation in 25 mM ammonium acetate (pH 7.5, Sigma-Aldrich, Germany) at 37°C with 0.6 U nuclease P1 from *P. citrinum* (Sigma-Aldrich, Germany), 0.2 U snake venom phosphodiesterase from *C. adamanteus* (Worthington, USA) and 2 U FastAP (Thermo Fisher Scientific, Germany), 10 U benzonase (Sigma-Aldrich, Germany), 200 ng Pentostatin (Sigma-Aldrich, Germany) and 500 ng Tetrahydrouridine (Merck-Millipore, Germany) were added. 5 μg RNA were analysed after digestion. The solvents consisted of 5 mM ammonium acetate buffer (pH 5.3; solvent A) and LC–MS grade acetonitrile (solvent B; Honeywell). The elution started with 100% solvent A, followed by a linear gradient to 8% solvent B at 10 min and 40% solvent B after 20 min. The flow rate was 0.35 ml/min on a Synergi Fusion column (4 μM particle size, 80 Å pore size, 250 × 2.0 mm from Phenomenex). ESI parameters were follow: gas temperature 300°C, gas flow 7 l/min, nebulizer pressure 60 psi, sheath gas temperature 400°C, sheath gas flow 12 l/min, capillary voltage 3000 V. The MS was operated in the positive ion mode using Agilent MassHunter software in the neutral loss scan mode with a loss of 132 Da (53).

### *In vitro* translation

Rabbit reticulocyte lysates, nuclease treated (54) (Promega) was programmed with 1 pmol mRNA in 50 μl final volume and incubated for 90 min at 30°C following the manufacturer's instructions. Aliquots of the reaction were mixed with non-reducing loading SDS-PAGE buffer (without β-mercaptoethanol) and loaded without pre-heating on an 10% SDS-polyacrylamide gel electrophoresis. Protein activity was directly assessed by in-gel fluorescence detection. The gel was placed in Typhoon scanner 9600 (GE Healthcare, United Kingdom) and fluorescence was detected by blue laser settings. Quantification of EGFP signal intensity was analysed by ImageJ normalized to the background. For radioactive labeling, the reaction was carried as previously described and supplemented by an amino acid mixture containing all the amino acids except methionine. To that, 20 mCi [35S]-methionine (PerkinElmer, Germany) was added and translation products were visualized by phosphorImaging.

## RESULTS

**Figure 1.**
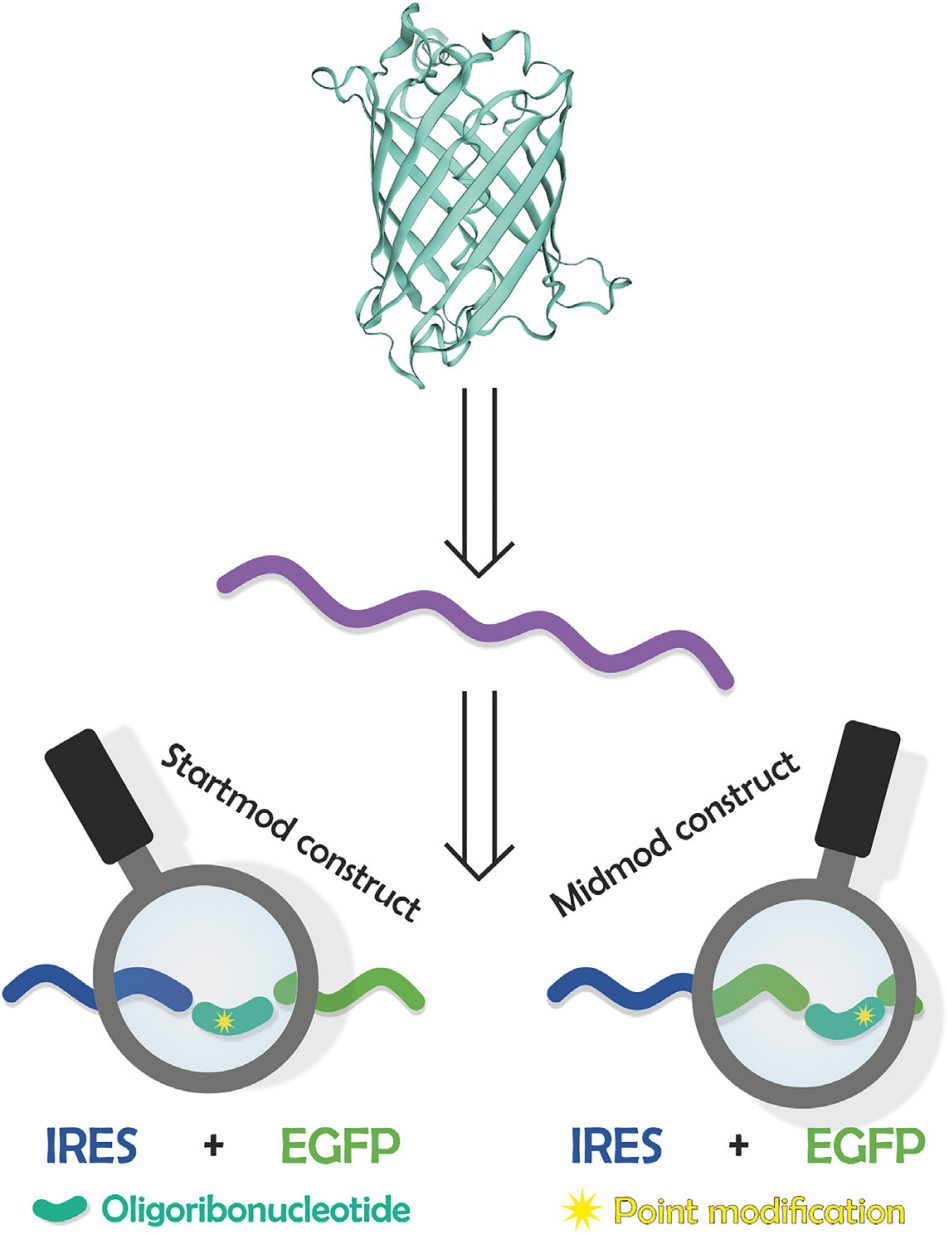
Retrosynthetic concept of point-modified mRNA. From target protein to RNA fragment level. The upper part shows the beta-barrel structure of the enhanced green fluorescent protein obtained from *in vitro* translation of a respective mRNA, shown purple in the middle part. The mRNA contained an EMCV-IRES moiety as translation initiation motif. The full-length mRNA was reassembled by splint ligation of three RNA Fragments: An IRES- (blue, fragment #1), a short synthetic oligonucleotide containing modified nucleotides (turquoise, fragment #2) and the 3′ fragment coding for the main part of EGFP- (green, fragment #3) fragment. The EGFP β-barrel was designed using ExPASy (107).

Similar to the development of synthetic approaches to therapeutic mRNA by *in vitro* transcription (IVT) (42,55–59), our approach to the synthesis of single-site-modified mRNA was relatively straightforward on paper (concept shown in Figure 1), but encountered numerous practical difficulties, especially with respect to yield, purification, and analysis. We here report the developed workaround solutions, leading to the characterization of the impact of ribose methylated nucleotides near the start codon on translation *in vitro*.

### Retrosynthetic mRNA breakdown and RNA ligation concept

As model reporter translation product, we focussed on the enhanced green fluorescent protein (EGFP) because of its robust folding and the resulting facile detection of functional protein. The chemical maturation of its fluorophore requires only ambient oxygen (60,61), while other reporter proteins require stoichiometric amounts of cofactors. For example, typical luciferases require stoichiometric amounts of ATP and luciferin in addition to ambient oxygen (62). Furthermore, proteins from the GFP family feature a barrel of beta-sheets around their fluorophore, which is stable enough that it can be post-translationally assembled from fragments (63) and which resists denaturation in SDS gels if sample heating is omitted prior to loading (64–66).

To circumvent incomplete cap synthesis on the mRNA, we turned to fusing an IRES upstream of the reporter, which previously had been found to ensure very efficient *in vitro* translation of the luciferase reporter gene (67). Therefore, we constructed a building block for site specifically modified EGFP mRNA with translation initiation *via* EMCV IRES. The latter is known to direct a robust translation level compared to other IRESs or capped mRNA (68).

For the introduction of a single modified nucleoside into what we termed point-modified mRNA, we retro-synthetically split its full-length sequence into three parts (Figure 1), to be joined by a three-way-one-pot splint ligation (41,69). To address the ensuing experimental prerequisites, we applied the following considerations:

In a first construct the modification was placed in a short RNA sequence corresponding to the middle of the overall RNA sequence, the short (‘middle’) fragment #2 being synthetically available by solid phase synthesis, thereby limiting its length to about 20–40 nucleotides (nts) (70). The remaining upstream (5′) fragment #1 and downstream (3′) fragment #3, because of their lengths, were synthesized by *in vitro* transcription with T7 RNA polymerase. For improved transcription yield, all constructs were designed such as to start with at least two G nucleotides at the 5′-end to promote efficient initiation by the polymerase (71). Typical ligation reactions contained equimolar amounts of the respective fragments and ∼0.98 equivalents of the splint cDNA. The similar size of the 5′ and 3′ fragments resulted in an mRNA almost twice as long as the individual fragments. Because of the length of the IRES, the synthetic modified RNA fragments came to bear at the beginning of the coding sequence. This construct was nicknamed ‘startmod’ construct. To demonstrate versatility, a second construct with a potential point modification in the middle of the coding sequence of EGFP named ‘midmod’ (see Figure 1) was conceived under similar requirements. In both cases, the mRNA product was substantially longer than the starting fragments, which allowed separation on a conventional low concentration denaturing PAGE, as well as by agarose gel electrophoresis.

**Figure 2.**
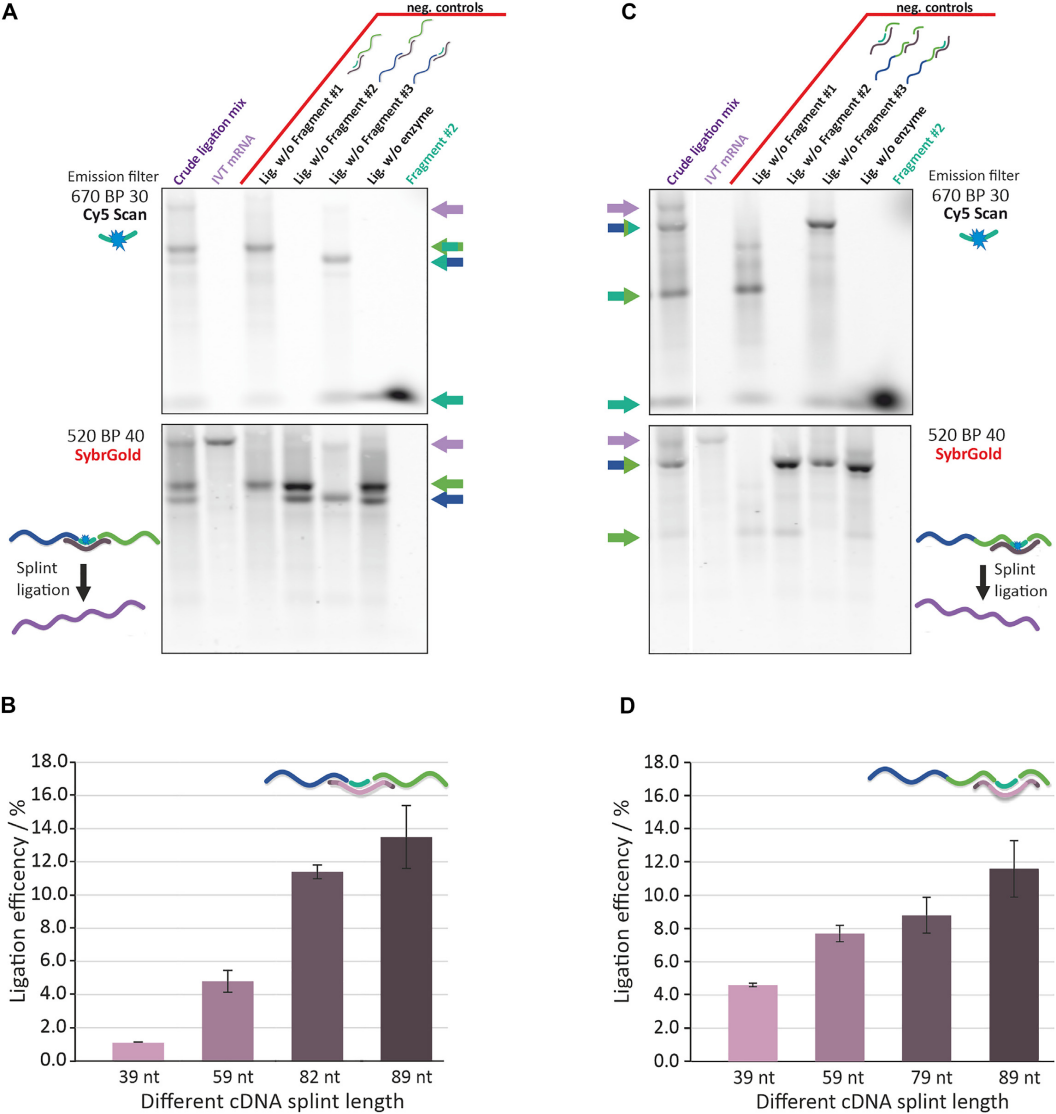
Results and validation of three-way-one-pot splint ligations. (**A**) Characterization of the *startmod* synthesis was carried out using a synthetic fragment #2 with a fluorescent Cy5-label as surrogate modification for orthogonal detection. The upper panel shows a fluorescence scan with Cy5 parameters of an agarose gel, therefore solely imaging RNAs that contain fragment #2. Identified RNAs are indicated by arrows to the right, which are color-coded as in Figure 1: purple for full-length mRNA, blue for RNAs containing fragment #1, turquoise for RNAs containing modified fragment #2, and green for RNAs containing fragment #3. The lower panel shows all RNAs as stained with SYBR^®^Gold in a fluorescence scan using the corresponding fluorescence scan parameters. A typical ligation reaction mixture was run in the left lane, next to an IVT mRNA. To the right of the red line were loaded various control reactions with omission of different RNAs or enzyme. Samples contained 400 ng RNA each. (**B**) length variation of the cDNA splint in *startmod* synthesis (**C**) Characterization of the *midmod* synthesis in analogy to (A). (**D**) length variation of the cDNA splint in *midmod* synthesis.

In a first optimization series, we used a fluorescent Cy5-labelled oligoribonucleotide as surrogate for the central ‘modified’ oligonucleotide in order to facilitate quantitative analytics. This enabled selective monitoring of the whereabouts of this central fragment, since the Cy5 emission is orthogonal to that of the SYBR^®^Gold stain used for the subsequent detection of all nucleic acids after electrophoresis. Figure 2A shows an agarose gel of a three-way-ligation using a cDNA splint that hybridizes all three RNA fragments in the correct order, thereby completely covering the length of the central Cy5-labeled #2 fragment. The corresponding Cy5-scan (upper panel) revealed three ligation products. Omission experiments shown on the right side of the gel allowed to identify the smaller two products as ligation products of fragments #1+#2, and fragments #2+#3, respectively. Comparison with the SYBR^®^Gold scan (displayed in the lower panel) shows that these ligations products are not effectively size-separated by agarose gel from the maternal fragments #2 and #3, respectively. The longest Cy5-containing ligation product was only generated in the presence of all three fragments, and its identity as full-length mRNA was confirmed in the SYBR^®^Gold scan (lower panel), where it is of identical length to an *in vitro* transcribed target-mRNA. Importantly, when fragment #2 was omitted, no ligation product of fragments #1 and #3 was detected, demonstrating that the synthesis of RNAs of relevant length always include the central fragment #2, i.e. fragments #1 and #3 were never joined by ligase.

The experiments shown in Figure 2 were performed under conditions previously optimized with respect to ligation temperature, duration, ligase concentration, and hybridization protocol. Overnight incubation at 16°C was a compromise with respect to low enzyme activity at lower temperatures and increased degradation at elevated temperatures and as such recapitulated our previous conclusions on the synthesis of short RNAs by the same method (74). The same holds true for various annealing protocols, ultimately showing no improvement over simple cooling at room temperature after heat denaturation.

Variation of the cDNA splint length over an accessible range of 40–90 nts revealed a significant increase in yield between 59 nts and 82 nts and moderate increase thereafter (Figure 2B). Of note, commercially accessible splint DNAs at higher lengths showed a significant drop in quality, especially homogeneity as judged by PAGE. In addition, we tested different parameters for the hybridization step of the 82 nts splint to the three fragments during the ligation reaction. We could not find significant differences for hybridization under controlled cooling (over 30 or 60 min), rapid cooling on ice, or at room temperature (Data not shown) for our constructs. The above is also in line with a longstanding experience in similar ligation experiments of smaller RNAs in our lab (41,69).

The above experiments spearheaded the synthesis of the ‘startmod’ construct but were also conducted for ‘midmod’ in an analogous fashion (Figure 2C). Results shown in Figure 2D show a similar dependence of ligation yield on cDNA length, albeit plateauing at lower values, despite the absence of strongly structured elements near the ligation site. Since these results suggested generally low yields in three-way-ligations with very long 5′-fragments, we made a ‘startmod’ derivative in which the long 5′-IRES containing fragment #1 was exchanged against a short 42mer of unrelated sequence. Indeed, the ligation yield improved significantly (Supplementary Figure S1). The lower yield in ‘startmod’ is suggestive of an influence of the highly structured IRES, yet the same does not apply to the situation in ‘midmod’. A final conclusion on a potential length-dependence of the ligation would have to include an in-depth study of RNA secondary structure, putting it clear out of scope of the present manuscript. We conclude that the splint was suitable for mediating a correctly oriented three-way-one-pot ligation, allowing to perform mRNA synthesis according to the concept of Figure 1 at different sites within a codon RNA.

To further demonstrate the applicability of the method we established two different constructs for splint ligation other than EGFP. These constructs encode in one case for mScarlet-I, a synthetic, monomeric, red fluorescent protein with enhanced maturation and eminent brightness compared to other mRFP proteins (72). The successful splint ligation is demonstrated in Supplementary Figure S7, which also shows the same series of controls as above. A third mRNA that was successfully synthesized codes for the Nsp13 protein from SARS-CoV-2 virus (Supplementary Figure S8).

Subsequent work was thus geared towards isolation, purification, characterization and functional analyses of full-length synthetic point-modified mRNA.

### Purification

**Figure 3.**
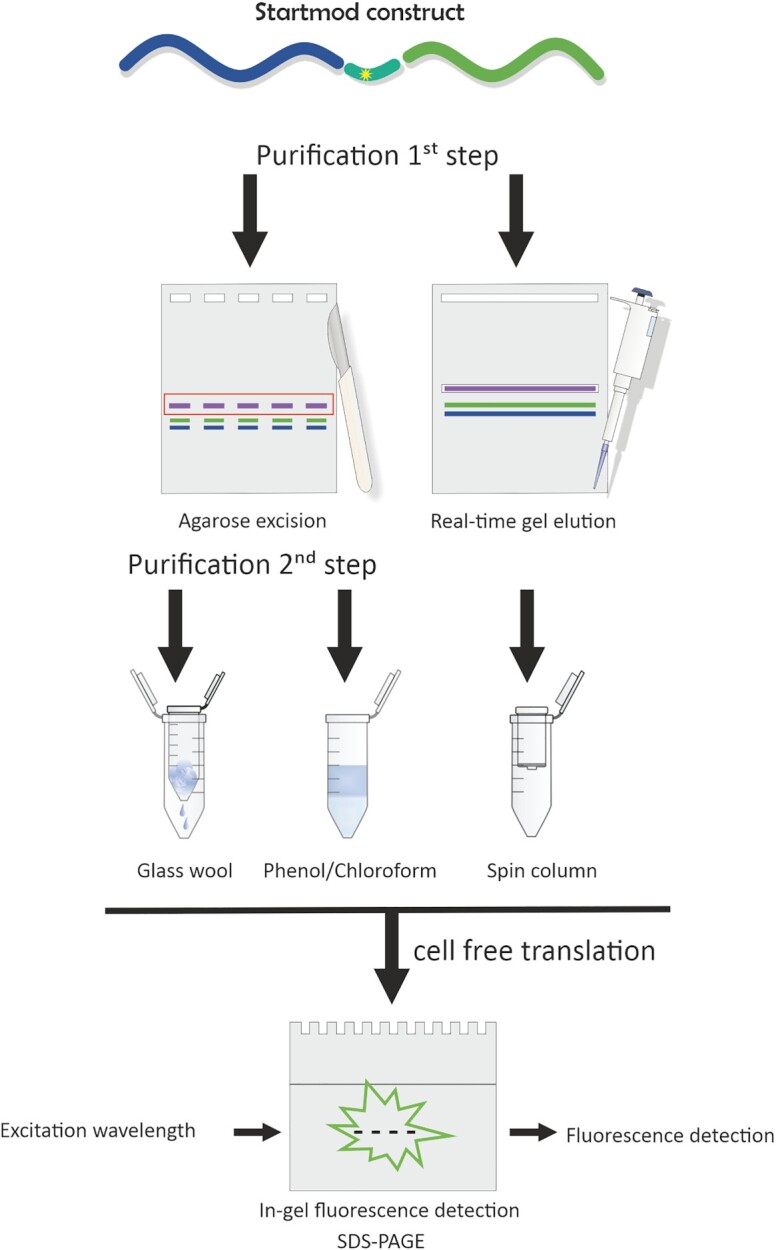
Overview of the tested purification methods. Left lane: The reaction mixture was applied to a 1% agarose gel (normal or low melting/gelling) and the desired band was excised. RNA was recovered either by glass wool filtration (left, normal agarose) or phenol-chloroform extraction (right, low melting/gelling agarose). Right lane: The reaction mixture was purified *via* real-time gel elution. Reaction mixtures were separated on a prestained 1% agarose gel and the desired band was identified by continuous visualization, and recovered while passing through an additional trough. Tus recovered RNA was filtered through a solid phase filter with a pore size of 0.2 μm. After cell free translation the translation efficiency was measured *via* in-gel fluorescence detection of EGFP.

While HPLC has been published as a viable method for purification of full-length, *in vitro* transcribed mRNA (42), none of our experimental setups for HPLC purification yielded reproducible and quantitative separation of full-length mRNA from the starting fragments (data not shown). Extraction from denaturing PAGE did allow separation but gave low and ill reproducible yields. Since the total length of the 1371 nts mRNA differs significantly from the length of the starting fragments (628 and 724 nts) and the analysis by agarose gel showed sufficient separation, we moved to mRNA purification by agarose gel (Figure 3).

A well-established method for DNA purification is extraction from low melting/gelling agarose gel (73–76). We adapted a protocol by Moore *et al.* with minor adjustments (Figure 3, middle, details in Materials and Methods) (49), finishing with a classical phenol/chloroform extraction. Working with this protocol, we noticed the repeated occurrence of solid material in redissolved RNA preparations after workup. This material, which occurred in amounts sufficiently abundant to disrupt subsequent HPLC–MS analysis, turned out to be residual agarose particles from the gel purification. In an attempt to reduce the amount of agarose particles, we excised RNA slices from conventional agarose gels and centrifuged through filtering glass wool (Figure 3, left). While the yields for both purification methods were comparable, significant reduction in agarose content was not achieved according to visual inspection of the pellets. While the isolated mRNA was intact, the cumulative experience over several dozen samples suggested that the most important parameter for optimum recovery and agarose content was precise excision of the band. This led us to conclude that (i) the ratio of RNA to gel slice volume is decisive, (ii) that a minimum contamination by agarose is unavoidable by this technique and (iii) that for small amounts of isolated mRNA, such as those issuing from ligation reactions, said ratio would likely be prohibitively unfavourable for practical application.

### Real-time gel elution

Based on the above observations, we set out to avoid excision of agarose slices entirely and turned to electroelution. Previous experience with the use of membranes for elution from PAGE slices (77–79) amounted to a conclusion similar to that drawn for agarose slices, namely that large volumes in relation to small amounts of RNA are prohibitive. In addition, electroelution devices and recent corresponding literature are sparse (80–82). To minimize elution volumes, we used a conventional ‘comb’ to create elution troughs in an agarose gel, similar to pockets used for sample loading. During electrophoresis, the RNA material proceeded into and through the trough and reentered the gel on the distal site, unless it was recovered by simple pipetting. Recovery of target RNA from the trough was thus contingent upon identification of the correct time frame during which the RNA traversed the trough. This was achieved by conducting electrophoresis in the presence of fluorescent staining dye (gel pre-staining with SYBR^®^Gold) and monitoring the RNA migration in real time using low energy UV-light for excitation. Technically, this was enabled by placing the electrophoresis chamber onto an illumination table (Supplementary Figure S2). In our hands, a DarkReader transilluminator, emitting blue visible and low energy UV light, worked well. A UV-transilluminator emitting only UV light (Hq, 254 nm/365 nm) was found inadequate, since the lucite walls of the agarose gel chamber acted as a UV-shield and thus prevented detection of the sample in reasonable quantities.

However, direct and continuous visualization with blue light enabled to recover RNA from the trough by stepwise pipetting. To this end, the voltage was cut when some target RNA had entered the trough, and after a first round of recovery, was re-started several times for 1–2 min until all material from the desired band had migrated into the collecting pocket. A video visualizing electrophoresis and recovery of a ligation reaction is provided with the supplementary information. To minimize RNA degradation, ultrapure water was used in all steps, including dilution of 10x TBE running buffer. To guard against residual unligated fragments in the trough pocket, the latter was rinsed several times with TBE buffer just before the full-length RNA migrated into the pocket. The eluate was spin-filtered using a pore size of 0.2 μm, precipitated, and further characterized. Typical elution yields varied between 15–40%, possibly depending on the length of the purified RNA. In combination with a ligation efficiency of ∼15%, this led to isolated yields of 3–7%.

### Blue light does not induce UV-like photodamage

**Figure 4.**
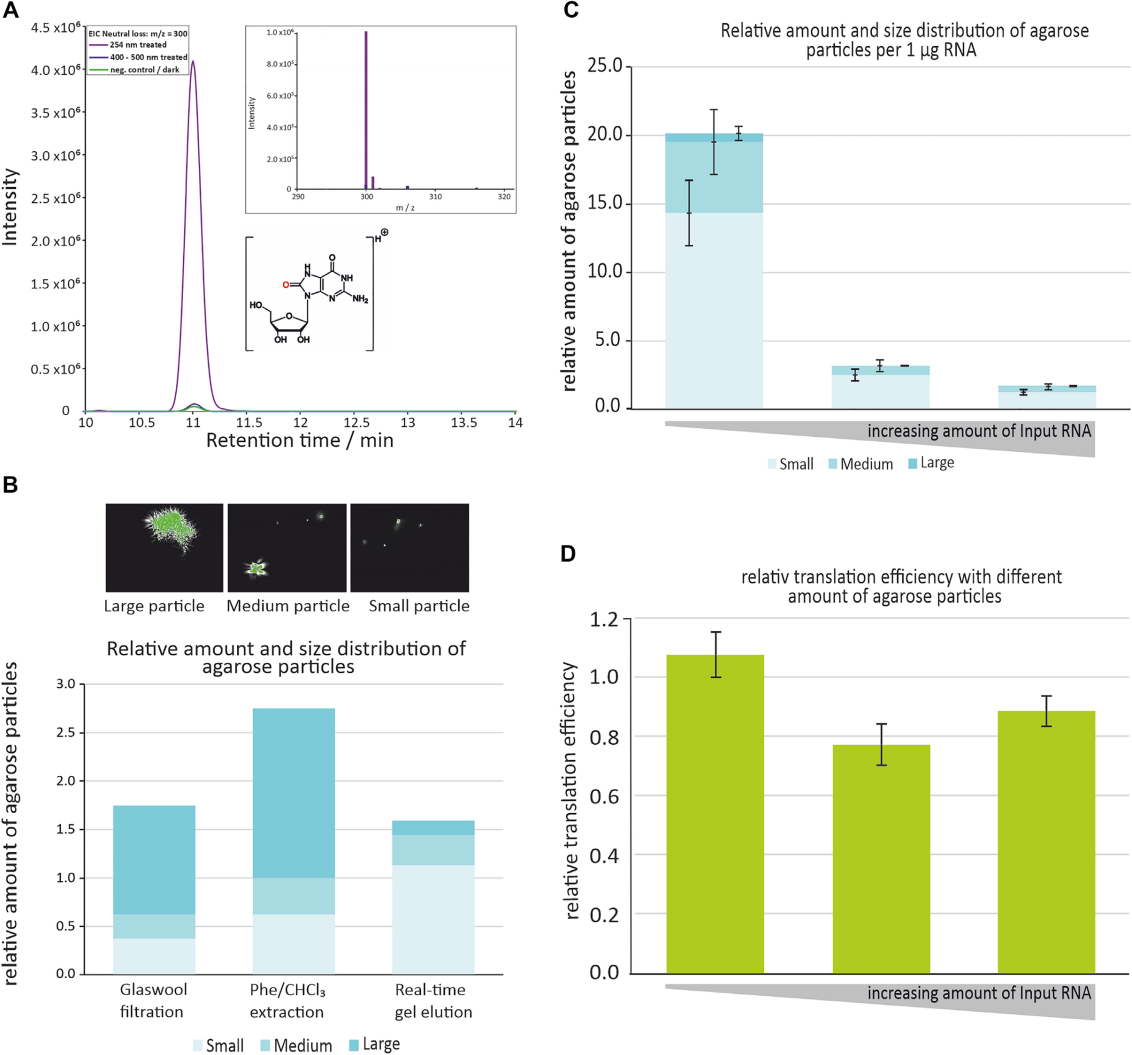
Validation of real-time elution. (**A**) Extracted Ion chromatogram of *m*/*z* 300 (8-Oxo-Guanosine, structure) after neutral loss of the ribose moiety (−132 Da). The inlet shows the mass spectrum (m/z scan) of 8-oxo-guanosine eluting at 11 min. A comparison is shown of RNA irradiated with no light (control, green trace), blue light (108), and UV-light (violet). The latter causes a strong increase in guanosine oxidation, while the signal after blue light irradiation is indistinguishable from the control. (**B**) Characterization of nanoparticles of different size by nanotracker. Relative amounts of small (light blue), medium (medium blue), and large particles (dark blue) were quantified, with real-time elution showing the lowest overall amount, as well as the lowest amount of large and medium sized particles. (**C**) agarose particle quantification in mRNA samples purified from different amounts of starting material (1.3, 4.5 and 16 μg), normalized to 1 μg isolated mRNA. The results demonstrate that larger amounts of starting material yield more favourable agarose content. (**D**) *In vitro* translation product of 1 pmol of each of the samples from (C) is of similar amplitude.

According to literature, exposure of nucleic acids to UV radiation may cause the formation of various damage products, including cyclobutane dimers, 8-oxo-guanosine, and hydrates of cytidine and uridine (83–87), although low energy UV light (UVA and UVB) was reported to promote formation of photoproducts with very low efficacy (84). To validate the use of extended irradiation (typically 30 min) of mRNA with blue light in this backdrop, we analysed *in vitro* transcribed mRNA with LC–MS after irradiation under different conditions. RNA was exposed for 120 min to either hard UV light (UVC, 254 nm), to the blue light from the DarkReader (400–500 nm), or to darkness at room temperature. Subsequently, samples were digested to nucleosides and analysed by LC–MS. A neutral-loss-scan (NLS, (53,88)) was implemented to detect all ribose-containing nucleoside species. Among the detected, a signal, that was identified as 8-oxo guanosine (8-OxoG) by its m/z value and comparison with authentic standard, was strongly increased after treatment with UVC (Figure 4A) but not after illumination with DarkReader blue light. We also detected very low signals for *m*/*z* values that correspond to other photoproducts from literature (e.g.) a hydrate of uridine (87), but none of these increased upon irradiation by either UVC or blue light. We conclude that the use of blue light for continuous illumination in real-time elution leaves the chemical structure of RNA intact.

### Agarose particle tracking

Given the previous observation of macroscopic amounts of agarose by simple visual inspection, it seemed reasonable to assume the presence of nanoscale particles as well. To assess the amount of agarose particles in an RNA preparation, a device (‘Nanotracker’) designed for measuring the size and amount of nanoparticles like e.g. liposomes (89) was employed. The instrument allows to track trajectories of particles in the applied sample by light scattering, *via* a microscope connected to a digital video camera. For nanoscale particles, the size of an observed particle can be estimated *via* its diffusion coefficient, and for larger particles, the instrument allows visual inspection of two-dimensional profiles in general. A sample was diluted to one millilitre and injected to the sample chamber, where it was manually inspected at random positions of the camera. The amount and size of agarose particles were characterized. The particles themselves were counted and sorted into three size categories, namely small, medium or large by digitally measuring the two-dimensional area in relation to a liposomal reference with a diameter of 130 nm.

Samples issuing from the different extraction methods were tested, when they involved agarose: low melting/gelling agarose followed by phenol/chloroform extraction, glass wool filtering of normal agarose, and real-time gel elution. Pellets resulting from precipitation of samples from the above methods were generally larger than typically expected from pure RNA, presumably due to a fair amount of agarose in all samples. The results given in Figure 4B show, that the phenol/chloroform extraction produced the most and largest particles, and the real-time gel elution the fewest and smallest with most of them featuring an approximated profile smaller than 1 μm^2^.

Given that the sample volume drawn from the trough was roughly equivalent among all samples claimed by real-time elution, we surmised that more concentrated samples would yield more RNA within the same amount drawn, and thus with a similar amount of agarose particles. This, in turn, would mean, that RNA recovered in low amounts would contain more agarose particles when normalized to the recovered RNA amount. In such cases, a high amount of particles per RNA molecule might turn out to be detrimental e.g. in translation. To address such a scenario, the degree of agarose particles in relation to RNA sample size was determined. A dilution series with samples containing 16, 4.5 and 1.3 μg RNA respectively was submitted to agarose electrophoresis followed by real-time elution with typical recovery rates around 15–20 %. The 1.3 μg was run 5 times to obtain enough recovered RNA. The agarose particle content corresponding of 1 μg mRNA was then compared among the samples. The results in Figure 4C do indeed confirm, that lower amounts of starting material lead to higher amounts of agarose particles when normalized to the total amount of mRNA. This, in turn, raised the concern that such high amounts of particles might compromise the *in vitro* mRNA translation in low yield samples. However, *in vitro* translation assays using 1 pmol of each mRNA preparation yielded comparable amounts of EGFP (Figure 4D). Even at highest agarose particle content, translation of mRNA synthesized by full-length IVT was equally efficient for material purified by real-time gel elution and by a silica column (*vide infra*), the latter being free of agarose.

We conclude that agarose particles in quantities relevant to real-time elution do not affect translation *in vitro*.

### Quality control of ligation products

For quality control of purification, we performed an analytical agarose gel showing that only minor amounts of starting material remained in the purified mRNA sample (Supplementary Figure S3, right). Since previous reports on mRNA synthesis (39) pointed out advantages of capillary electrophoresis, such as high sample throughput with low sample amounts in a short time (2 minutes) we intermittently switched our analysis of mRNA samples to the TapeStation system from Agilent. This device allows automated capillary electrophoresis of nucleic acid samples (RNA and DNA) to control their quality mainly used for quality control prior to sequencing (90). The internal size standard, added to every sample, reportedly allows a precise size indication and concentration determination of the sample. However, when comparing aliquot analyses of identical sample side-by-side, quantification results of full-length mRNA versus ligation fragments were incoherent between the two methods (Supplementary Figure S3, left). In analyses of samples from other research lines in our lab on the same TapeStation instrument, we repeatedly observed strong variation of migration behaviour of long fragments, causing us to err on the side of caution by continuing to rely on agarose gel for mRNA analysis.

**Figure 5.**
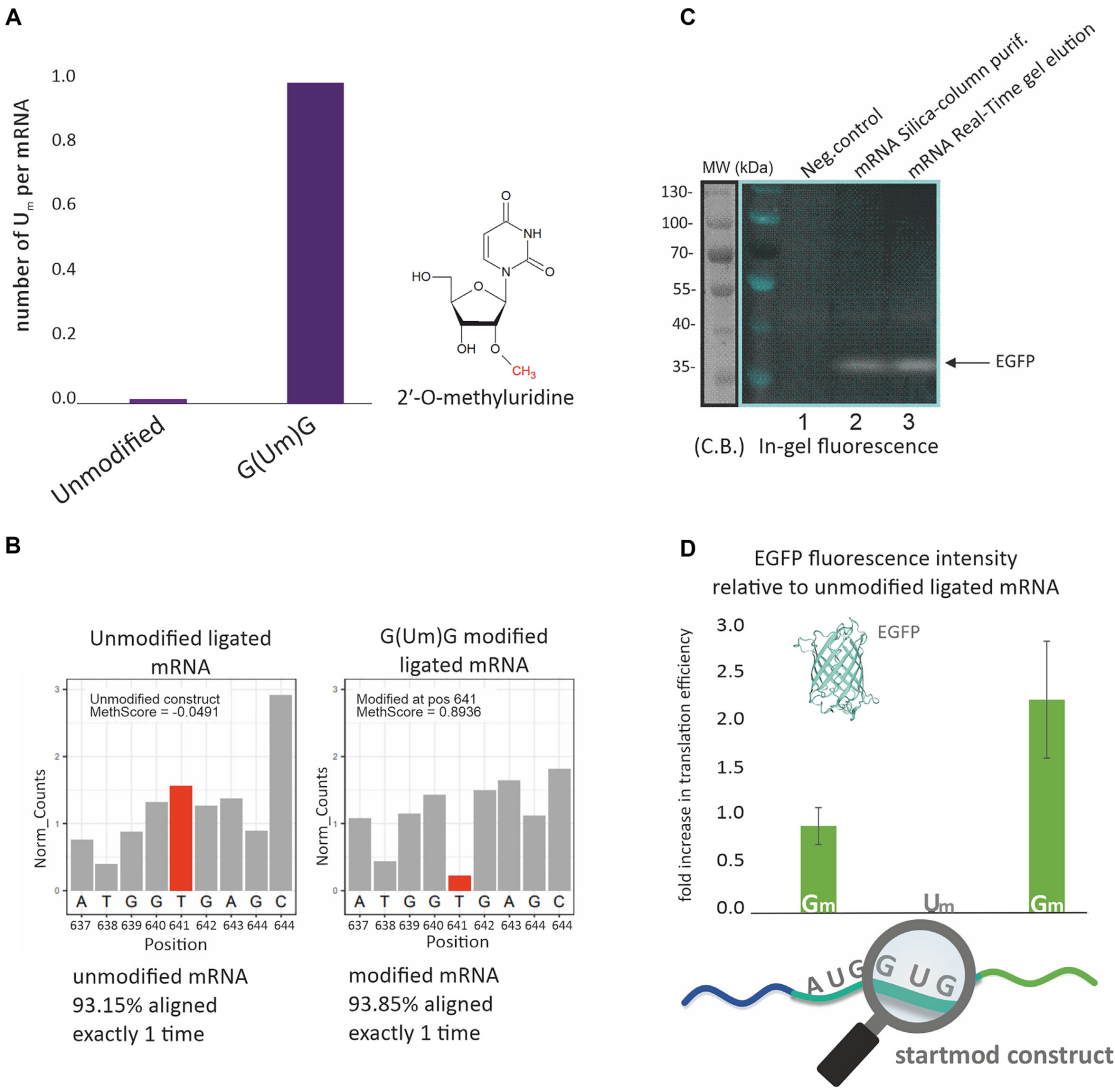
Quality control and functional assay for point-modified mRNAs. (**A**) Absolute LC-MS/MS quantification of the number of 2′-*O*-methylated Uridines per IRES-EGFP mRNA molecule after ligation of the site specifically modified Oligonucleotide to the two unmodified IVT fragments. An unmodified ligation reference showed no detectable Um. The real-time gel eluted ligation samples were digested to the nucleoside level and analysed *via* LC-MS/MS. For absolute quantification 13C, stable isotope-labelled nucleosides from *S. cerevisiae* were used as an internal standard and mixed in an equal amount with the analysed sample, as technical duplicates. (**B**) RiboMethSeq protection profiles for unmodified and 2′-*O*-methylated constructs. Normalized RiboMethSeq cleavage profiles around the position 641 are shown for the unmodified and modified construct. Position of modification is shown by a red bar. The MethScores (representing modification level) for position 641 are given for each construct. (**C**) Monitoring protein expression by in-gel fluorescence detection. Translation of EGFP mRNA purified mRNAs either by silica-column or eluted by Real-Time in treated RRL. After translation, an aliquot from the reaction mixture was loaded on 10% SDS-PAGE, and fluorescence was measured by in-gel scanning using the corresponding excitation/emission wavelength settings. On the side, the weight ladder was also detected using a red laser and after staining with Coomassie Blue (C.B.). (**D**) Translation efficiency of point-modified EGFP mRNA. The fluorescence intensity was determined relative to the non-modified ligated mRNA. Errors bars indicate ± SD of independent triplicates.

The sequence of the purified ligated mRNA was verified by Illumina sequencing (Supplementary Figure S4). Indeed, as surmised from the pertinent control experiments in Figure 2, the splint DNA ensured ligation of the three fragments in correct order, and no indication of further, non-templated ligation events on either the 5′- or the 3′-end. Correct incorporation of the modified nucleotide was verified by LC-MS. Based on previous reports (35,37), ribose methylation at the second position within a codon was expected to significantly impede protein translation. In the scope of our investigations, this applied to a U_m_ in in the middle of the second codon (GUG, pos. 640–642), which therefore warranted particular scrutiny. We used LC–MS analysis to quantify the amount of U_m_ on nucleoside level of the digested mRNA and found almost exactly one U_m_ per mRNA for the construct 2′-*O*-methylated (Figure 5A), but no significant amount of U_m_ in the unmodified ligation product used as control. We furthermore used our ribose-methylation specific Illumina RNAseq derivative named RiboMethSeq (91,92), to demonstrate the correct positioning of said U_m_ (Figure 5B), and the adjacent ribose methylations (pos. 640 and 642) in the respective constructs (Supplementary Figure S5).

### Functional assay for purified mRNA

As outlined above, the enhanced green fluorescent protein (EGFP), an optimized version of the original GFP (93) was chosen as reporter because the straightforward read-out of its fluorescence provides a measure for of the efficacy of ribosomal biosynthesis of functional protein, without the need of adding further cofactors. Numerous tools are available for manipulation and quantification *e.g*. of fusion proteins containing EGFP as a localization tag (94). EGFP-coding mRNA purified by real-time gel elution was subjected to *in vitro* translation in Nulease-treated rabbit reticulocyte lysate (RRL). Interestingly, the folded fluorescent protein was found stable under the rather harsh conditions of a conventional SDS-PAGE when sample heating prior to loading was omitted (64–66).

Based on these prerequisites, we explored the possibility of designing an assay for quantification of *in vitro* translation *via* in-gel detection of EGFP. As a first key parameter for robustness, we established a plateau value for EGFP maturation time. Samples of 1 pmol of full-length mRNA were subjected to an *in vitro* translation assay for different periods of time, and the resulting proteins were separated on an a conventional SDS-PAGE as described above. The gel was then scanned for green fluorescence. Supplementary Figure S6A shows the resulting imaged bands, whose intensity, when plotted as a function of incubation time, reached a plateau after 90 minutes. This indicates that chosen assay time at 90 min is robust with respect to any further maturation events. Supplementary Figure S6B shows results from a titration of mRNA concentration. At the manufacturer's recommended concentration of 36 nM mRNA (marke din by a red circle), EGFP translation reached a plateau, possibly masking potential further increases in translation efficiency relative to normal. We therefore placed our standard concentration at 20 nM (black arrow), i.e. near the middle of the dynamic range, to better capture deviations relative to normal.

A gel displaying fluorescent bands obtained under these conditions is shown in Figure 5C. Lanes 2 and 3 show protein fluorescence after translation of full-length IVT mRNA purified by real-time gel elution versus purification by silica-column next to a negative control without mRNA. On the same gel, the protein ladder can also be detected by changing the settings of the excitation and emission wavelengths (Only excitation by red laser). The molecular weight of the EGFP is ∼27 kDa, even though the observed band exhibited a migration at odds with the calculated molecular weight. This migration behaviour is well described in conditions which are only mildly denaturing, including non-reducing conditions and omission of sample pre-heating to preserve the structure of the GFP (64,95,96). In this case, this reflects that the expressed protein is intact and correctly folded. Posterior Coomassie staining accurately confirmed the ladder and EFGP bands, effectively validating the continued use of in-gel detection by fluorescence without additional staining. Examples for further validation, namely *in vitro* translation in the presence of ^35^S-labeled methionine, were conducted on modified mRNA (*vide infra*). Of note, neither Coomassie nor ^35^S imaging revealed any indication of the presence of abortive translation products.

### Functionality of point-modified mRNA

With all the prerequisites addressed, we applied the method to its originally intended purpose, namely to test the impact of single, site-specific modifications in a long mRNA on its translation. The objective of the ‘startmod’ construct was to synthesize a point-modified IRES-EGFP mRNA carrying ribose methylations (2′-OCH_3_ modifications) just downstream of the start codon, i.e. concerning the first codon decoded by a ‘conventional’ (i.e. non-initiator) tRNA. Using a more rudimentary *in vitro* translation system, Choi *et al.* (37) had reported a strong negative effect of ribose methylation at the context second position of a codon, whereas ribose methylations at positions 1 and 3 were found not to be detrimental. Here, analogous ribose methylation patterns were synthesized and analysed in the codon just downstream of the start AUG codon in our IRES-EGFP construct. Figure 5D shows translation efficacies as a function of the position of the ribose methylation. The results indeed document a rather drastic negative effect of a GUmG codon, while GmUG was moderately affected and GUGm even showed an increase in functional protein. Of note, the differences in translation as measured by in-gel fluorescence were adequately recapitulated in the simultaneously effect radioactive labelling of the synthetized protein by S35 (Supplementary Figure S6C). The results of our *in vitro* translation are quite plausible in the context of the previously mentioned findings (35). They also correlate with the effect observed in the previously mentioned mechanistic studies on the single molecule level (37).

## DISCUSSION

Recent advanced detection methods (97) led to numerous reports on post-transcriptional modification mapping in coding sequences (98–100). This invariably raises the question about the influence of mRNA modification on protein synthesis. Given that enhancement versus impediment of mRNA translation may hinge on a single methyl group (35), research groups from academia and industry alike have developed a vested interest in methods to approach this question experimentally. Given that synthetic mRNAs have been commercialized, optimization of features such as UTRs (101,102), codon usage (103), cap structure chemistry, and non-canonical nucleosides is inevitable. Hence, a closer look at point modification, expanding on current methods (104–106) is easily anticipated. The Erlacher group has recently made important inroads in this respect, both in the synthesis of mRNAs by splint ligation, and by an innovative purification approach using poly-dT-coated beads for isolation of polyA-mRNA (35). A particularly impressive feat was their transfection of thus point-modified mRNA into HEK cells, resulting in efficient translation. The three-way-one-pot splint ligation presented here, which we already used for smaller RNAs (69), now provides access to mRNAs much longer than those cited above. These mRNAs may carry the point modification at essentially any site, provided that the starting material and full-length product can be separated on agarose gel. The characteristic structure-function pattern of ribose methylation at positions one through three of a codon (Figure 5) faithfully recapitulates findings from three studies, one on a short model mRNA (37), the other using a longer mRNA in a bacterial *in vitro* translation system (38), and the third actually upon transfection into eukaryotic cells (35) and thus provides mutual confirmation of the validity of the experimental systems.

The key development here was clearly the efficient and clean recovery from the agarose gel. Once conceived, the concept seems straightforward, to the point of almost being self-evident; it did, however, take us several years to come up with this method after all others proved too inefficient for the production of mRNA above the nanogram regime. Since we could show that agarose particles, which invariably contaminate the eluted mRNA, do not negatively affect mRNA translation in the applied quantities, we are confident, that they will be even less of a problem in upscaled ligation reactions.

## DATA AVAILABILITY

ExPASy is the SIB Bioinformatics Resource Portal which provides access to scientific databases and software tools (https://www.expasy.org/).

## Supplementary Material

gkac719_Supplemental_Files
