# Supplementary material for: Synthesis of point-modified mRNA
Source: Nucleic Acids Res. 2022 Sep 5;50(20):e115. doi: 10.1093/nar/gkac719 (PMC9723659; doi:10.1093/nar/gkac719)

Supplementary information

Synthesis of point-modified mRNA

Jasmin Hertler[1,*], Kaouthar Slama[1,*], Benedikt Schober[1], Zeynep Özrendeci[1], Virginie Marchand[2], Yuri Motorin[2,3] and Mark Helm[1,#]

[1] Institute of Pharmaceutical and Biomedical Sciences, Johannes Gutenberg-Universität, Staudinger Weg 5, D-55128 Mainz, Germany.
[2]IMoPA UMR7365 CNRS-UL, BioPole Université de Lorraine, Vandœuvre-lès-Nancy, [3]France.Epitranscriptomics and RNA Sequencing (EpiRNA-Seq) Core Facility, UMS2008 IBSLor (CNRS-UL)/US40 (INSERM), Université de Lorraine, Vandœuvre-lès-Nancy, France.

[*]The authors wish it to be known that, in their opinion, the first 2 authors should be regarded as joint First Authors

[#]To whom correspondence should be addressed. Tel: +49 6131 392 5731; Fax: +49 6131 392 0373; Email: mhelm@uni-mainz.de

Figure S1

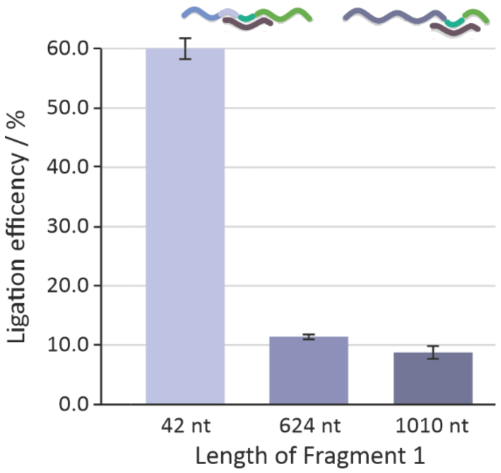

Figure S1 Ligation efficiencies of different systems varying the length of the fragments at the 5’ end (indicated by the schematic figures on the top).

Figure S2

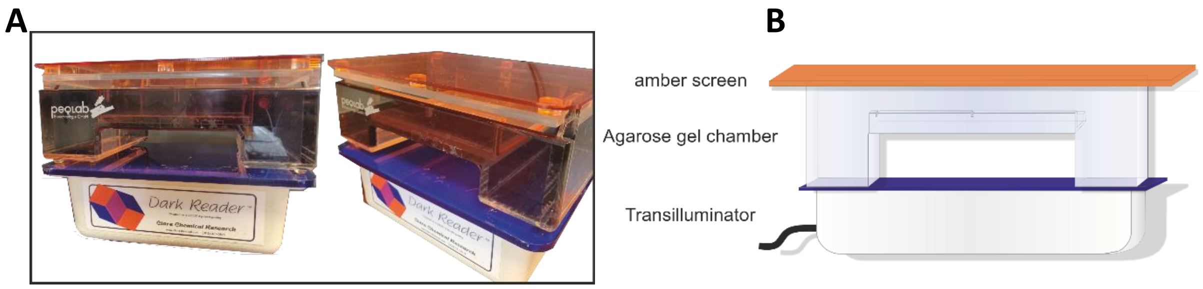

Figure S2 Photograph (**A**) and schematic setup (**B**) of the Real-Time gel purification: The Agarose gel chamber is placed on top of the Transilluminator which used visible blue light instead of UV light as excitation source. To visualize the RNA during running of the gel an amber screen is needed. Background from photograph removed.

Figure S3

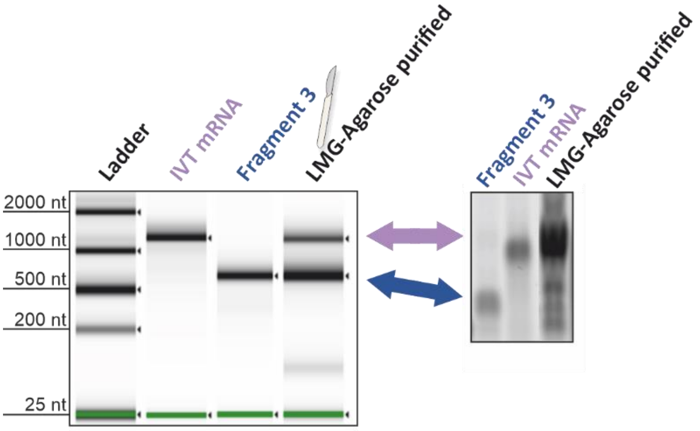

Figure S3 Tapestation analysis was compared to analysis by 1 % agarose gel. For both methods the same samples were used. On the left the results from the tapestation run of IVT mRNA (pos. control, light purple), Fragment 3 of the 5' construct (blue) and a ligation sample after purification by low melting agarose are shown. The agarose gel was stained with SybrGold. For Gel analysis 100 ng pure RNA was used.

Figure S4

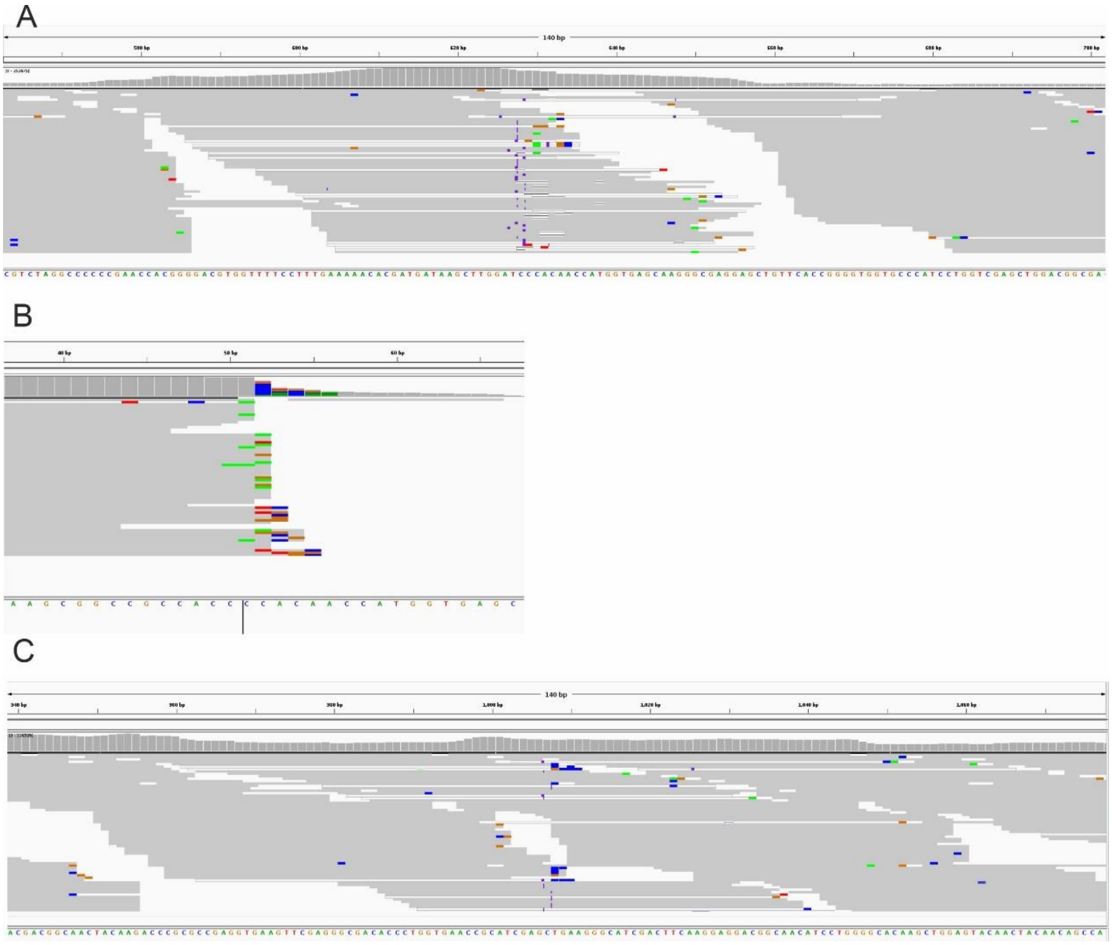

Figure S4. Read alignment views by IGV software. (A) The top panel summarized the reads alignment as a coverage plot. Nucleotides with mismatch frequencies higher than 20% are colored in green (Adenosine mismatch), blue (Cytidine), orange (Guanosine) and/or green (Thymidine). The middle panel shows reads alignment at nucleotide resolution. Purple boxes represent insertions and black stripes gaps. The bottom panel displays the reference sequence. Alignment view at the insertion point of startmod construct. Multiple insertions can be seen. **(B)** View of reads aligned to the 3' end of the ligated mRNA extended by the middle fragment. The 3' end mRNA sequence is located at the left side from the black line; while the middle fragment sequence is located at the right side from it. **(C)** Alignment reads of the midmod construct.

Figure S5

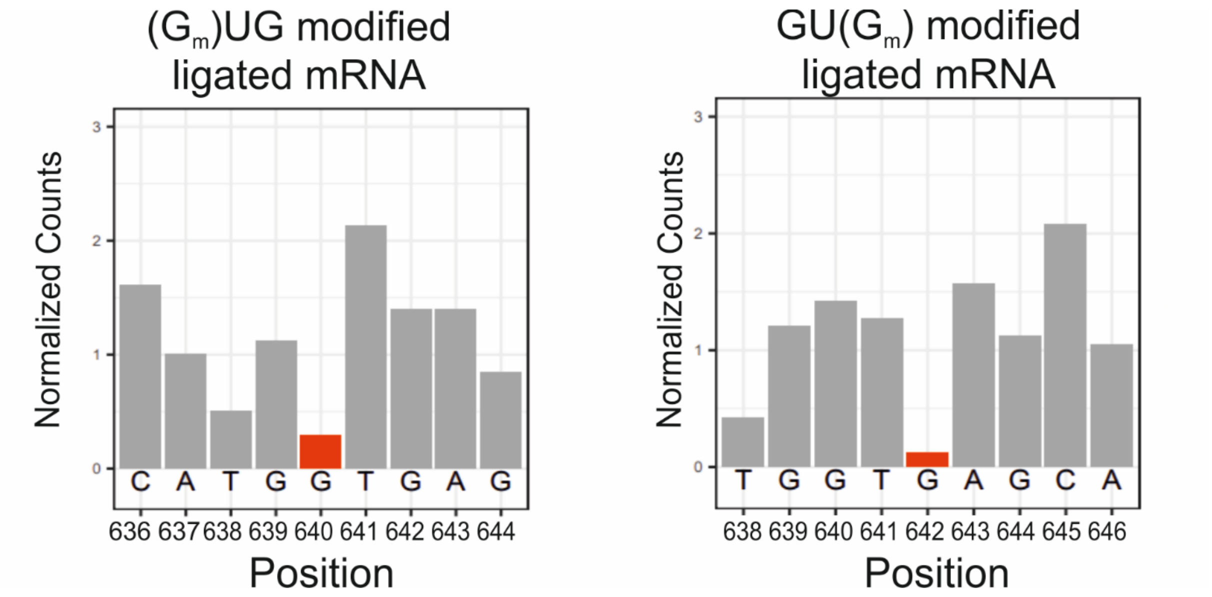

Figure S5. Analysis of 2’-O-Me point-modified mRNA. The RiboMethSeq protection profiles for point-modified mRNA containing 2´O-methylation at position 640 and position 642 are given for each construct.

Figure S6

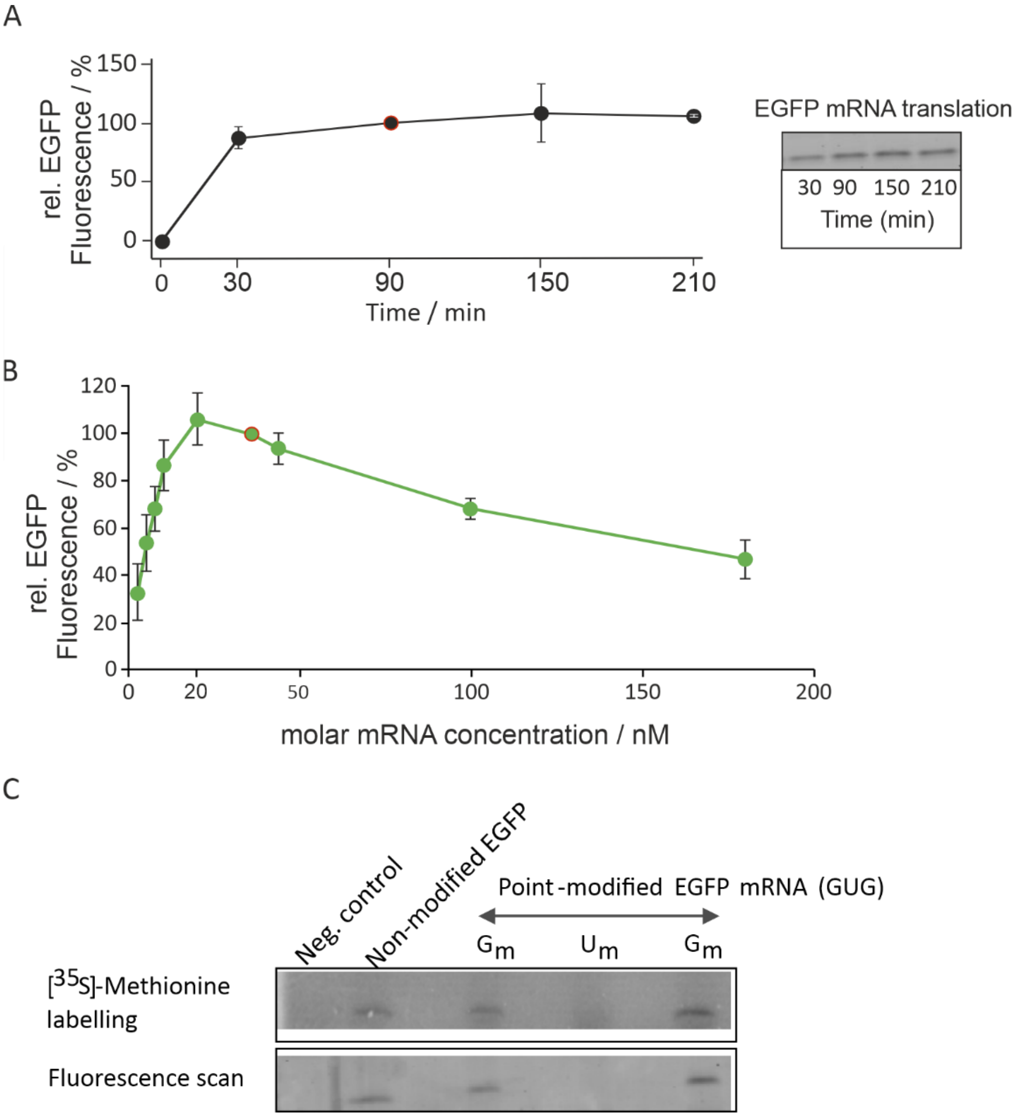

Figure S6. Assay design for In-gel fluorescence. **(A)** Protein synthesis was assessed at the indicated incubation times, using nuclease-treated RRL. Aliquots were analyzed on 10% SDS-PAGE and scanned for EGFP fluorescence by in-gel detection. Results from technical triplicates. Normalized to 90 min (indicated with red circle). **(B)** Nuclease treated RRL was programmed with increasing concentration of mRNA (2.5, 5, 7.5, 10, 20, 36, 44, 100 and 180 nM, green dots). After translation, aliquots were separated on 10 % SDS-PAGE, and EGFP fluorescence was in-gel detected by blue laser settings (488 nm/ 520 nm excitation/emission wavelengths) normalized to 36 nM (amount recommended by the manufacturer, indicated with red circle). Results from biological triplicates. **(C)** *In vitro* translation of single nucleoside-modified mRNA. [^{35}S]-methionine-labeled EGFP translated proteins were applied for fluorescence detection, followed by exposure to a phosphor imaging plate and identification.

Figure S7

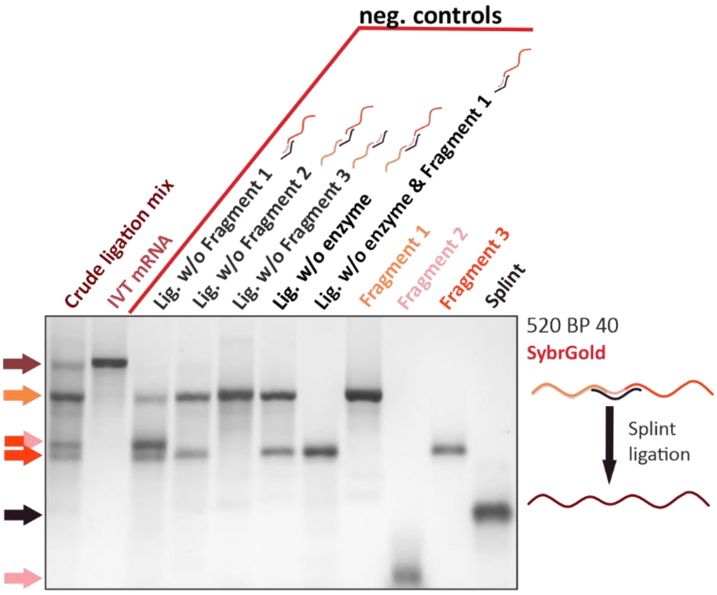

Figure S7. Result of mScarlet-I Splint ligation. This gel demonstrated the applicability of the splint ligation towards other constructs than EGFP. Identified RNAs are highlighted by arrows on the right in the respective colour. Full length mRNA is coloured in bordeaux red, orange indicates fragment #1, modified fragment #2 is represented as pink while fragment #3 is bright red. Additionally, the DNA splint is coloured in black. The ligation reaction mixture was load next to the control IVT mRNA followed by several control reactions. 2 % Agarose gel, 10V/cm, samples contained 200 ng RNA stained with SYBR®Gold.

Figure S8

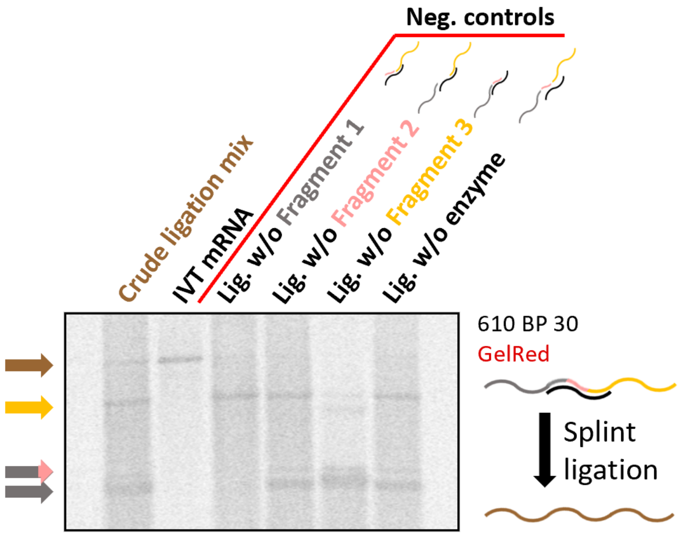

Figure S8. Result of SARS-CoV-2 Nsp13 Splint ligation. This gel demonstrated the applicability of the splint ligation towards other constructs than EGFP. Full length mRNA is coloured in brown, dark grey indicates fragment #1, modified fragment #2 is represented as pink while fragment #3 is gold. The ligation reaction mixture was loaded next to the control IVT mRNA followed by several control reactions. 1% Agarose gel stained with GelRed, 10V/cm, samples contained 1000 ng RNA.

Sequence of the used Oligonucleotides:

Unmodified fragment #2 (startmod construct): 5’-CCACAACCAUGGUGAGCAA-3’

Codon which was modified by single 2’ O-ribose methylation is underlined. Either G_m (on first or third codon position) or U_m was used.

Unmodified fragment #2 (midmod construct): 5’-UGAAGGGCAUCGACUUCAA-3’

Modified fragment #2 (mScarlet-I construct): 5’- CGGUUGUACCCCGAGGACG_mGCGUGCUGAAG-3’

Modified fragment #2 (SARS-CoV-2 Nsp13 construct): 5’- AAUUACAU_mCUUUCAUGGGAAGUU-3’

Sequence of IVT Fragment #1 for startmod construct containing EMCV IRES

5’GGGCGAAUUGGGUACCGGGCCCCCCCUCGAGGUCAUCGAAUUCCGCCCCUCUCCCUCCCCCCCCCCUAA
CGUUACUGGCCGAAGCCGCUUGGAAUAAGGCCGGUGUGCGUUUGUCUAUAUGUUAUUUUCCACCAUAU
UGCCGUCUUUUGGCAAUGUGAGGGCCCGGAAACCUGGCCCUGUCUUCUUGACGAGCAUUCCUAGGGGU
CUUUCCCCUCUCGCCAAAGGAAUGCAAGGUCUGUUGAAUGUCGUGAAGGAAGCAGUUCCUCUGGAAGCU
UCUUGAAGACAAACAACGUCUGUAGCGACCCUUUGCAGGCAGCGGAACCCCCCACCUGGCGACAGGUGCC
UCUGCGGCCAAAAGCCACGUGUAUAAGAUACACCUGCAAAGGCGGCACAACCCCAGUGCCACGUUGUGAG
UUGGAUAGUUGUGGAAAGAGUCAAAUGGCUCUCCUCAAGCGUAUUCAACAAGGGGCUGAAGGAUGCCC
AGAAGGUACCCCAUUGUAUGGGAUCUGAUCUGGGGCCUCGGUGCACAUGCUUUACAUGUGUUUAGUCG
AGGUUAAAAAAACGUCUAGGCCCCCCGAACCACGGGGACGUGGUUUUCCUUUGAAAAACACGAUGAUAA
GCUUGGAUC-3’

Sequence of IVT Fragment #1 for midmod construct containing EMCV IRES and part of EGFP coding region (start codon in bold)

5’GGGCGAAUUGGGUACCGGGCCCCCCCUCGAGGUCAUCGAAUUCCGCCCCUCUCCCUCCCCCCCCCCUAA
CGUUACUGGCCGAAGCCGCUUGGAAUAAGGCCGGUGUGCGUUUGUCUAUAUGUUAUUUUCCACCAUAU
UGCCGUCUUUUGGCAAUGUGAGGGCCCGGAAACCUGGCCCUGUCUUCUUGACGAGCAUUCCUAGGGGU
CUUUCCCCUCUCGCCAAAGGAAUGCAAGGUCUGUUGAAUGUCGUGAAGGAAGCAGUUCCUCUGGAAGCU
UCUUGAAGACAAACAACGUCUGUAGCGACCCUUUGCAGGCAGCGGAACCCCCCACCUGGCGACAGGUGCC
UCUGCGGCCAAAAGCCACGUGUAUAAGAUACACCUGCAAAGGCGGCACAACCCCAGUGCCACGUUGUGAG
UUGGAUAGUUGUGGAAAGAGUCAAAUGGCUCUCCUCAAGCGUAUUCAACAAGGGGCUGAAGGAUGCCC
AGAAGGUACCCCAUUGUAUGGGAUCUGAUCUGGGGCCUCGGUGCACAUGCUUUACAUGUGUUUAGUCG
AGGUUAAAAAAACGUCUAGGCCCCCCGAACCACGGGGACGUGGUUUUCCUUUGAAAAACACGAUGAUAA
GCUUGGAUCCCACAACC**AUG**GUGAGCAAGGGCGAGGAGCUGUUCACCGGGGUGGUGCCCAUCCUGGUC
GAGCUGGACGGCGACGUAAACGGCCACAAGUUCAGCGUGUCCGGCGAGGGCGAGGGCGAUGCCACCUAC
GGCAAGCUGACCCUGAAGUUCAUCUGCACCACCGGCAAGCUGCCCGUGCCCUGGCCCACCCUCGUGACCA
CCCUGACCUACGGCGUGCAGUGCUUCAGCCGCUACCCCGACCACAUGAAGCAGCACGACUUCUUCAAGUC
CGCCAUGCCCGAAGGCUACGUCCAGGAGCGCACCAUCUUCUUCAAGGACGACGGCAACUACAAGACCCGC
GCCGAGGUGAAGUUCGAGGGCGACACCCUGGUGAACCGCAUCGAGC-3’

Sequence of IVT Fragment #3 for startmod construct containing most of the coding region of EGFP (stop codon in bold)

5’GGGCGAGGAGCTGTTCACCGGGGTGGTGCCCATCCTGGTCGAGCTGGACGGCGACGTAAACGGCCACAAG
TTCAGCGTGTCCGGCGAGGGCGAGGGCGATGCCACCTACGGCAAGCTGACCCTGAAGTTCATCTGCACCACC
GGCAAGCTGCCCGTGCCCTGGCCCACCCTCGTGACCACCCTGACCTACGGCGTGCAGTGCTTCAGCCGCTACCC
CGACCACATGAAGCAGCACGACTTCTTCAAGTCCGCCATGCCCGAAGGCTACGTCCAGGAGCGCACCATCTTC
TTCAAGGACGACGGCAACTACAAGACCCGCGCCGAGGTGAAGTTCGAGGGCGACACCCTGGTGAACCGCATC
GAGCTGAAGGGCATCGACTTCAAGGAGGACGGCAACATCCTGGGGCACAAGCTGGAGTACAACTACAACAGC
CACAACGTCTATATCATGGCCGACAAGCAGAAGAACGGCATCAAGGTGAACTTCAAGATCCGCCACAACATCG
AGGACGGCAGCGTGCAGCTCGCCGACCACTACCAGCAGAACACCCCCATCGGCGACGGCCCCGTGCTGCTGC
CCGACAACCACTACCTGAGCACCCAGTCCGCCCTGAGCAAAGACCCCAACGAGAAGCGCGATCACATGGTCCT
GCTGGAGTTCGTGACCGCCGCCGGGATCACTCTCGGCATGGACGAGCTGTACAAG**TAA**AGCGGCCGCCACC
GC-3’

Sequence of IVT Fragment #3 for midmod construct containing second part of the coding region of EGFP (stop codon in bold)

5’GGAGGACGGCAACAUCCUGGGGCACAAGCUGGAGUACAACUACAACAGCCACAACGUCUAUAUCAUGG
CCGACAAGCAGAAGAACGGCAUCAAGGUGAACUUCAAGAUCCGCCACAACAUCGAGGACGGCAGCGUGCA
GCUCGCCGACCACUACCAGCAGAACACCCCCAUCGGCGACGGCCCCGUGCUGCUGCCCGACAACCACUACC
UGAGCACCCAGUCCGCCCUGAGCAAAGACCCCAACGAGAAGCGCGAUCACAUGGUCCUGCUGGAGUUCGU
GACCGCCGCCGGGAUCACUCUCGGCAUGGACGAGCUGUACAAG**UAA**AGCGGCCGCCACCGC-3’

cDNA splint for startmod construct

5’-TGGGCACCACCCCGGTGAACAGCTCCTCGCCCTTGCTCACCATGGTTGTGGCAAGCTTATCATCGTGTTTTTC
AAAGG-3’

cDNA splint for midmod construct

5’AGCTTGTGCCCCAGGATGTTGCCGTCCTCCTTGAAGTCGATGCCCTTCAGCTCGATGCGGTTCACCAGGGTG
TCGCCCT-3’

Full-length mRNA sequence with modifiable regions from startmod constructs underlined (with modified codon in red+underlined). Start and stop codon in bold.

5’GGGCGAAUUGGGUACCGGGCCCCCCCUCGAGGUCAUCGAAUUCCGCCCCUCUCCCUCCCCCCCCCCUAA
CGUUACUGGCCGAAGCCGCUUGGAAUAAGGCCGGUGUGCGUUUGUCUAUAUGUUAUUUUCCACCAUAU
UGCCGUCUUUUGGCAAUGUGAGGGCCCGGAAACCUGGCCCUGUCUUCUUGACGAGCAUUCCUAGGGGU
CUUUCCCCUCUCGCCAAAGGAAUGCAAGGUCUGUUGAAUGUCGUGAAGGAAGCAGUUCCUCUGGAAGCU
UCUUGAAGACAAACAACGUCUGUAGCGACCCUUUGCAGGCAGCGGAACCCCCCACCUGGCGACAGGUGCC
UCUGCGGCCAAAAGCCACGUGUAUAAGAUACACCUGCAAAGGCGGCACAACCCCAGUGCCACGUUGUGAG
UUGGAUAGUUGUGGAAAGAGUCAAAUGGCUCUCCUCAAGCGUAUUCAACAAGGGGCUGAAGGAUGCCC
AGAAGGUACCCCAUUGUAUGGGAUCUGAUCUGGGGCCUCGGUGCACAUGCUUUACAUGUGUUUAGUCG
AGGUUAAAAAAACGUCUAGGCCCCCCGAACCACGGGGACGUGGUUUUCCUUUGAAAAACACGAUGAUAA
GCUUGGAUCCCACAACC**AUG**GUGAGCAAGGGCGAGGAGCUGUUCACCGGGGUGGUGCCCAUCCUGGUC
GAGCUGGACGGCGACGUAAACGGCCACAAGUUCAGCGUGUCCGGCGAGGGCGAGGGCGAUGCCACCUAC
GGCAAGCUGACCCUGAAGUUCAUCUGCACCACCGGCAAGCUGCCCGUGCCCUGGCCCACCCUCGUGACCA
CCCUGACCUACGGCGUGCAGUGCUUCAGCCGCUACCCCGACCACAUGAAGCAGCACGACUUCUUCAAGUC
CGCCAUGCCCGAAGGCUACGUCCAGGAGCGCACCAUCUUCUUCAAGGACGACGGCAACUACAAGACCCGC
GCCGAGGUGAAGUUCGAGGGCGACACCCUGGUGAACCGCAUCGAGCUGAAGGGCAUCGACUUCAAGGAG
GACGGCAACAUCCUGGGGCACAAGCUGGAGUACAACUACAACAGCCACAACGUCUAUAUCAUGGCCGACA
AGCAGAAGAACGGCAUCAAGGUGAACUUCAAGAUCCGCCACAACAUCGAGGACGGCAGCGUGCAGCUCGC
CGACCACUACCAGCAGAACACCCCCAUCGGCGACGGCCCCGUGCUGCUGCCCGACAACCACUACCUGAGCA
CCCAGUCCGCCCUGAGCAAAGACCCCAACGAGAAGCGCGAUCACAUGGUCCUGCUGGAGUUCGUGACCGC
CGCCGGGAUCACUCUCGGCAUGGACGAGCUGUACAAG**UAA**AGCGGCCGCCACCGC-3’

Full length mScarlet-I Sequence

5’GGCAAUCCAAGUCCUAACCCCCAACCCAACUCUAGCCCAUCCGGCCAACCAACCUACCAUGGUGAGCAAG
GGCGAGGCAGUGAUCAAGGAGUUCAUGCGGUUCAAGGUGCACAUGGAGGGCUCCAUGAACGGCCACGA
GUUCGAGAUCGAGGGCGAGGGCGAGGGCCGCCCCUACGAGGGCACCCAGACCGCCAAGCUGAAGGUGAC
CAAGGGUGGCCCCCUGCCCUUCUCCUGGGACAUCCUGUCCCCUCAGUUCAUGUACGGCUCCAGGGCCUUC
AUCAAGCACCCCGCCGACAUCCCCGACUACUAUAAGCAGUCCUUCCCCGAGGGCUUCAAGUGGGAGCGCG
UGAUGAACUUCGAGGACGGCGGCGCCGUGACCGUGACCCAGGACACCUCCCUGGAGGACGGCACCCUGA
UCUACAAGGUGAAGCUCCGCGGCACCAACUUCCCUCCUGACGGCCCCGUAAUGCAGAAGAAGACAAUGGG
CUGGGAAGCGUCCACCGAGCGGUUGUACCCCGAGGACGGCGUGCUGAAGGGCGACAUUAAGAUGGCCCU
GCGCCUGAAGGACGGCGGCCGCUACCUGGCGGACUUCAAGACCACCUACAAGGCCAAGAAGCCCGUGCAG
AUGCCCGGCGCCUACAACGUCGACCGCAAGUUGGACAUCACCUCCCACAACGAGGACUACACCGUGGUGG
AACAGUACGAACGCUCCGAGGGCCGCCACUCCACCGGCGGCAUGGACGAGCUGUACAAGUGACAAUUAAC
AGCCAAACUAG3’

Sequence of IVT Fragment #1 for mScarlet-I construction

5’GGCAAUCCAAGUCCUAACCCCCAACCCAACUCUAGCCCAUCCGGCCAACCAACCUACCAUGGUGAGCAAG
GGCGAGGCAGUGAUCAAGGAGUUCAUGCGGUUCAAGGUGCACAUGGAGGGCUCCAUGAACGGCCACGA
GUUCGAGAUCGAGGGCGAGGGCGAGGGCCGCCCCUACGAGGGCACCCAGACCGCCAAGCUGAAGGUGAC
CAAGGGUGGCCCCCUGCCCUUCUCCUGGGACAUCCUGUCCCCUCAGUUCAUGUACGGCUCCAGGGCCUUC
AUCAAGCACCCCGCCGACAUCCCCGACUACUAUAAGCAGUCCUUCCCCGAGGGCUUCAAGUGGGAGCGCG
UGAUGAACUUCGAGGACGGCGGCGCCGUGACCGUGACCCAGGACACCUCCCUGGAGGACGGCACCCUGA
UCUACAAGGUGAAGCUCCGCGGCACCAACUUCCCUCCUGACGGCCCCGUAAUGCAGAAGAAGACAAUGGG
CUGGGAAGCGUCCACCGAG3’

Sequence of IVT Fragment #3 for mScarlet-I construction

5’GGCGACAUUAAGAUGGCCCUGCGCCUGAAGGACGGCGGCCGCUACCUGGCGGACUUCAAGACCACCUA
CAAGGCCAAGAAGCCCGUGCAGAUGCCCGGCGCCUACAACGUCGACCGCAAGUUGGACAUCACCUCCCAC
AACGAGGACUACACCGUGGUGGAACAGUACGAACGCUCCGAGGGCCGCCACUCCACCGGCGGCAUGGACG
AGCUGUACAAGUGACAAUUAACAGCCAAACUAG3’

cDNA splint for mScarlet-I construct

CCGTCCTTCAGGCGCAGGGCCATCTTAATGTCGCCCTTCAGCACGCCGTCCTCGGGGTACAACCGCTCGGTGG
ACGCTTCCCAGCCCATTGTCTTCTTCT

Full length SARS-Cov-2 Nsp13 Sequence

5’GCUGUUGGGGCUUGUGUUCUUUGCAAUUCACAGACUUCAUUAAGAUGUGGUGCUUGCAUACGUAGA
CCAUUCUUAUGUUGUAAAUGCUGUUACGACCAUGUCAUAUCAACAUCACAUAAAUUAGUCUUGUCUGU
UAAUCCGUAUGUUUGCAAUGCUCCAGGUUGUGAUGUCACAGAUGUGACUCAACUUUACUUAGGAGGUA
UGAGCUAUUAUUGUAAAUCACAUAAACCACCCAUUAGUUUUCCAUUGUGUGCUAAUGGACAAGUUUUU
GGUUUAUAUAAAAAUACAUGUGUUGGUAGCGAUAAUGUUACUGACUUUAAUGCAAUUGCAACAUGUG
ACUGGACAAAUGCUGGUGAUUACAUUUUAGCUAACACCUGUACUGAAAGACUCAAGCUUUUUGCAGCA
GAAACGCUCAAAGCUACUGAGGAGACAUUUAAACUGUCUUAUGGUAUUGCUACUGUACGUGAAGUGCU
GUCUGACAGAGAAUUACAUCUUUCAUGGGAAGUUGGUAAACCUAGACCACCACUUAACCGAAAUUAUGU
CUUUACUGGUUAUCGUGUAACUAAAAACAGUAAAGUACAAAUAGGAGAGUACACCUUUGAAAAAGGUG
ACUAUGGUGAUGCUGUUGUUUACCGAGGUACAACAACUUACAAAUUAAAUGUUGGUGAUUAUUUUGU
GCUGACAUCACAUACAGUAAUGCCAUUAAGUGCACCUACACUAGUGCCACAAGAGCACUAUGUUAGAAU
UACUGGCUUAUACCCAACACUCAAUAUCUCAGAUGAGUUUCUAGCAAUGUUGCAAAUUAUCAAAAGGU
UGGUAUGCAAAAGUAUUCUACACUCCAGGGACCACCUGGUACUGGUAAGAGUCAUUUUGCUAUUGGCC
UAGCUCUCUACUACCCUUCUGCUCGCAUAGUGUAUACAGCUUGCUCUCAUGCCGCUGUUGAUGCACUAU
GUGAGAAGGCAUUAAAAUAUUUGCCUAUAGAUAAAUGUAGUAGAAUUAUACCUGCACGUGCUCGUGUA
GAGUGUUUUGAUAAAUUCAAAGUGAAUUCAACAUUAGAACAGUAUGUCUUUUGUACUGUAAAUGCAU
UGCCUGAGACGACAGCAGAUAUAGUUGUCUUUGAUGAAAUUUCAAUGGCCACAAAUUAUGAUUUGAGU
GUUGUCAAUGCCAGAUUACGUGCUAAGCACUAUGUGUACAUUGGCGACCCUGCUCAAUUACCUGCACCA
CGCACAUUGCUAACUAAGGGCACACUAGAACCAGAAUAUUUCAAUUCAGUGUGUAGACUUAUGAAAACU
AUAGGUCCAGACAUGUUCCUCGGAACUUGUCGGCGUUGUCCUGCUGAAAUUGUUGACACUGUGAGUGC
UUUGGUUUAUGAUAAUAAGCUUAAAGCACAUAAAGACAAAUCAGCUCAAUGCUUUAAAAUGUUUUAUA
AGGGUGUUAUCACGCAUGAUGUUUCAUCUGCAAUUAACAGGCCACAAAUAGGCGUGGUAAGAGAAUUC
CUUACACGUAACCCUGCUUGGAGAAAAGCUGUCUUUAUUUCACCUUAUAAUUCACAGAAUGCUGUAGCC
UCAAAGAUUUUGGGACUACCAACUCAAACUGUUGAUUCAUCACAGGGCUCAGAAUAUGACUAUGUCAUA
UUCACUCAAACCACUGAAACAGCUCACUCUUGUAAUGUAAACAGAUUUAAUGUUGCUAUUACCAGAGCA
AAAGUAGGCAUACUUUGCAUAAUGUCUGAUAGAGACCUUUAUGACAAGUUGCAAUUUACAAGUCUUGA
AAUUCCACGUAGGAAUGUGGCAACUUUACAA-3’

Sequence of IVT Fragment #1 for SARS-Cov-2 Nsp13 construction

5’GCUGUUGGGGCUUGUGUUCUUUGCAAUUCACAGACUUCAUUAAGAUGUGGUGCUUGCAUACGUAGA
CCAUUCUUAUGUUGUAAAUGCUGUUACGACCAUGUCAUAUCAACAUCACAUAAAUUAGUCUUGUCUGU
UAAUCCGUAUGUUUGCAAUGCUCCAGGUUGUGAUGUCACAGAUGUGACUCAACUUUACUUAGGAGGUA
UGAGCUAUUAUUGUAAAUCACAUAAACCACCCAUUAGUUUUCCAUUGUGUGCUAAUGGACAAGUUUUU
GGUUUAUAUAAAAAUACAUGUGUUGGUAGCGAUAAUGUUACUGACUUUAAUGCAAUUGCAACAUGUG
ACUGGACAAAUGCUGGUGAUUACAUUUUAGCUAACACCUGUACUGAAAGACUCAAGCUUUUUGCAGCA
GAAACGCUCAAAGCUACUGAGGAGACAUUUAAACUGUCUUAUGGUAUUGCUACUGUACGUGAAGUGCU
GUCUGACAGAG-3’

Sequence of IVT Fragment #3 for SARS-Cov-2 Nsp13 construction

5’GGUAAACCUAGACCACCACUUAACCGAAAUUAUGUCUUUACUGGUUAUCGUGUAACUAAAAACAGUAA
AGUACAAAUAGGAGAGUACACCUUUGAAAAAGGUGACUAUGGUGAUGCUGUUGUUUACCGAGGUACAA
CAACUUACAAAUUAAAUGUUGGUGAUUAUUUUGUGCUGACAUCACAUACAGUAAUGCCAUUAAGUGCA
CCUACACUAGUGCCACAAGAGCACUAUGUUAGAAUUACUGGCUUAUACCCAACACUCAAUAUCUCAGAU
GAGUUUUCUAGCAAUGUUGCAAAUUAUCAAAAGGUUGGUAUGCAAAAGUAUUCUACACUCCAGGGACC
ACCUGGUACUGGUAAGAGUCAUUUUGCUAUUGGCCUAGCUCUCUACUACCCUUCUGCUCGCAUAGUGU
AUACAGCUUGCUCUCAUGCCGCUGUUGAUGCACUAUGUGAGAAGGCAUUAAAAUAUUUGCCUAUAGAU
AAAUGUAGUAGAAUUAUACCUGCACGUGCUCGUGUAGAGUGUUUUGAUAAAUUCAAAGUGAAUUCAAC
AUUAGAACAGUAUGUCUUUUGUACUGUAAAUGCAUUGCCUGAGACGACAGCAGAUAUAGUUGUCUUUG
AUGAAAUUUCAAUGGCCACAAAUUAUGAUUUGAGUGUUGUCAAUGCCAGAUUACGUGCUAAGCACUAU
GUGUACAUUGGCGACCCUGCUCAAUUACCUGCACCACGCACAUUGCUAACUAAGGGCACACUAGAACCAG
AAUAUUUCAAUUCAGUGUGUAGACUUAUGAAAACUAUAGGUCCAGACAUGUUCCUCGGAACUUGUCGG
CGUUGUCCUGCUGAAAUUGUUGACACUGUGAGUGCUUUGGUUUAUGAUAAUAAGCUUAAAGCACAUAA
AGACAAAUCAGCUCAAUGCUUUAAAAUGUUUUAUAAGGGUGUUAUCACGCAUGAUGUUUCAUCUGCAA
UUAACAGGCCACAAAUAGGCGUGGUAAGAGAAUUCCUUACACGUAACCCUGCUUGGAGAAAAGCUGUCU
UUAUUUCACCUUAUAAUUCACAGAAUGCUGUAGCCUCAAAGAUUUUGGGACUACCAACUCAAACUGUUG
AUUCAUCACAGGGCUCAGAAUAUGACUAUGUCAUAUUCACUCAAACCACUGAAACAGCUCACUCUUGUA
AUGUAAACAGAUUUAAUGUUGCUAUUACCAGAGCAAAAGUAGGCAUACUUUGCAUAAUGUCUGAUAGA
GACCUUUAUGACAAGUUGCAAUUUACAAGUCUUGAAAUUCCACGUAGGAAUGUGGCAACUUUACAA-3’

cDNA splint for SARS-Cov-2 Nsp13 construct

TTTCGGTTAAGTGGTGGTCTAGGTTTACCAACTTCCCATGAAAGATGTAATTCTCTGTCAGACAGCACTTCACGT
ACAGT

Vectormap pUC57 (pDNA origin of IVT fragments)

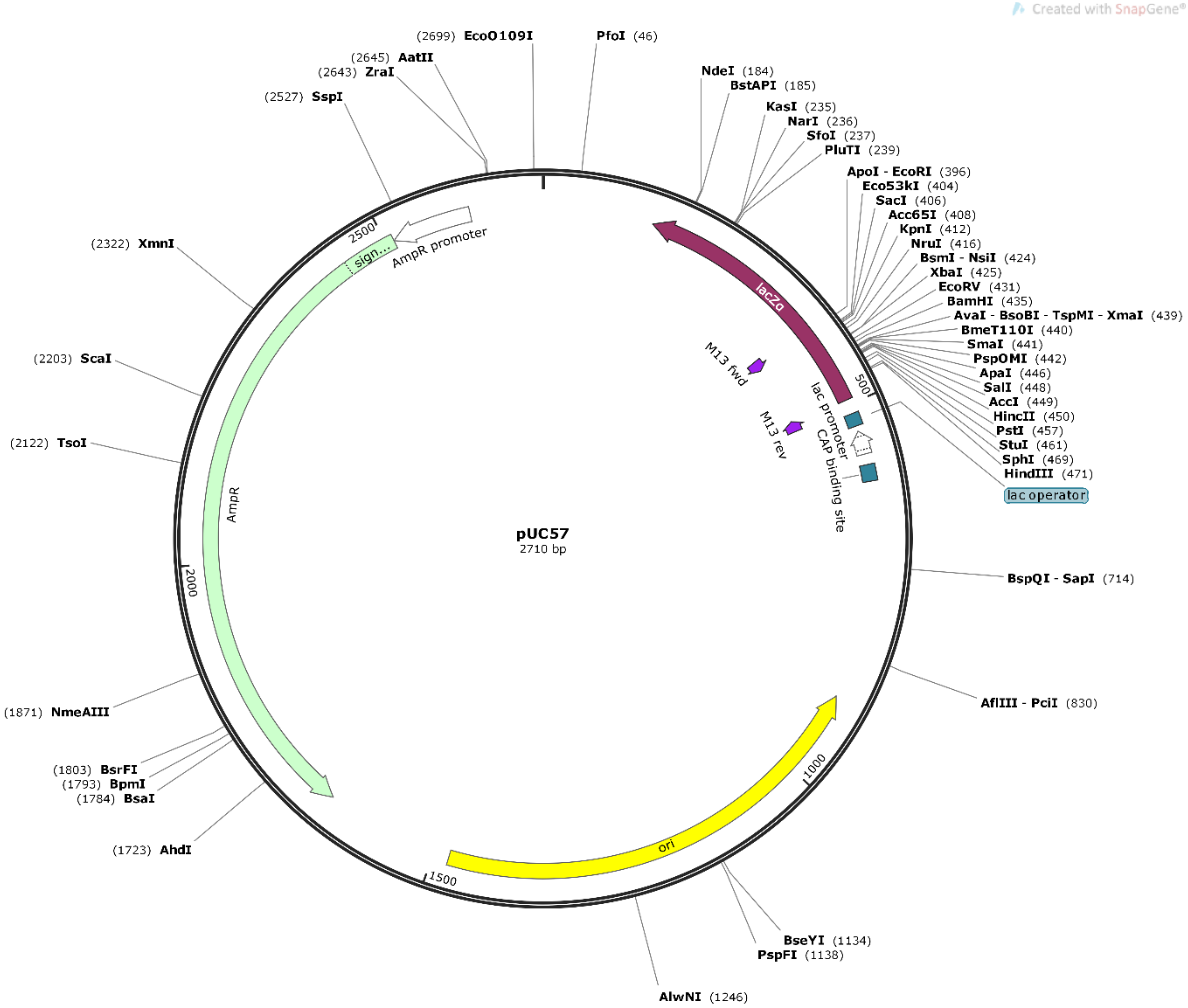

Supplement: gkac719_Supplemental_Files [file gkac719_supplemental_files.zip › Supplementary Information_026.pdf]
