# Supplementary material for: Synthesis of point-modified mRNA
Source: Nucleic Acids Res. 2022 Sep 5;50(20):e115. doi: 10.1093/nar/gkac719 (PMC9723659; doi:10.1093/nar/gkac719)
Supplement: gkac719_Supplemental_Files [file gkac719_supplemental_files.zip › Video caption.pdf]

Video Caption

Synthesis of point-modified mRNA

Jasmin Hertler[1,*], Kaouthar Slama[1,*], Benedikt Schober[1], Zeynep Özrendeci[1], Virginie Marchand[2], Yuri Motorin[2,3] and Mark Helm[1,#]

[1] Institute of Pharmaceutical and Biomedical Sciences, Johannes Gutenberg-Universität, Staudinger Weg 5, D-55128 Mainz, Germany.
[2]IMoPA UMR7365 CNRS-UL, BioPole Université de Lorraine, Vandœuvre-lès-Nancy,
[3]France.Epitranscriptomics and RNA Sequencing (EpiRNA-Seq) Core Facility, UMS2008 IBSLor (CNRS-UL)/US40 (INSERM), Université de Lorraine, Vandœuvre-lès-Nancy, France.

[*]The authors wish it to be known that, in their opinion, the first 2 authors should be regarded as joint First Authors

[#]To whom correspondence should be addressed. Tel: +49 6131 392 5731; Fax: +49 6131 392 0373; Email: mhelm@uni-mainz.de

The video, attached in an additional supplementary file, demonstrates the method of real-time gel elution. The complete volume of a ligation reaction was applied to the upper trough of an 1 % agarose gel and after intercalating with SybrGoldTM, the RNA was followed in real-time. After separation into the different RNA species the brightest band, representing the unligated RNA fragments, was eluted from the gel before the ligation product was collected with an 1000 µL pipette.
